# An Adenoviral‐Vectored Tp0326 Vaccine Elicits Robust Functional Antibodies to Prevent *Treponema pallidum* Dissemination in a Rabbit Model

**DOI:** 10.1002/advs.78078

**Published:** 2026-09-28

**Authors:** Yinbo Jiang, Xinyuan Li, Lixia Huang, Xiangcai Zhang, Wujian Ke

**Affiliations:** ^1^ Dermatology Hospital Southern Medical University Guangzhou Guangdong China; ^2^ Hospital for Skin Diseases Institute of Dermatology Chinese Academy of Medical Sciences and Peking Union Medical College Nanjing Jiangsu China; ^3^ National Center for Sexually Transmitted Disease Control Chinese Center for Disease Control and Prevention Nanjing Jiangsu China; ^4^ School of Public Health Nanjing Medical University Nanjing Jiangsu China; ^5^ The First School of Clinical Medicine Southern Medical University Guangzhou Guangdong China

**Keywords:** BamA, function antibody, rabbit infectivity test, syphilis vaccine, β‐barrel protein

## Abstract

Vaccination can prevent congenital infections by inducing antibodies that block transplacental pathogen transmission. For syphilis, which is caused by *Treponema pallidum (T. pallidum)*, no vaccine is yet available to halt its vertical spread to fetuses. Here, we designed an adenovirus‐vectored vaccine expressing the *T. pallidum* outer membrane protein TP0326 and evaluated its immunogenicity and protective efficacy in animal models. The vaccine elicited robust humoral and Th2‑biased cellular immune responses in both mice and rabbits, with resulting antibodies exhibiting potent anti‑adhesion, opsonophagocytic, and bactericidal activities. In rabbits challenged with high‐ or low‐dose *T. pallidum*, vaccination significantly reduced lesion severity and tissue bacterial burden, while enhancing local phagocytic responses. Intramuscular immunization promoted early opsonophagocytosis and restricted pathogen dissemination by rabbit infectivity test. These findings demonstrate that the TP0326 based adenovirus vaccine induces functional antibody‑mediated immunity and supports its further development as a candidate vaccine for preventing congenital syphilis.

## Graphical abstract

Vaccination promotes cellular infiltration into primary lesions and effectively inhibits *T. pallidum* dissemination to distal organs. Notably, transfer of lesion tissue from immunized animals fails to establish infection in naive recipients, confirming specific protective immunity. The mechanism involves local antibody induction at primary sites, facilitating direct pathogen killing and enhancing spirochete clearance via macrophage phagocytosis, collectively preventing secondary infections.

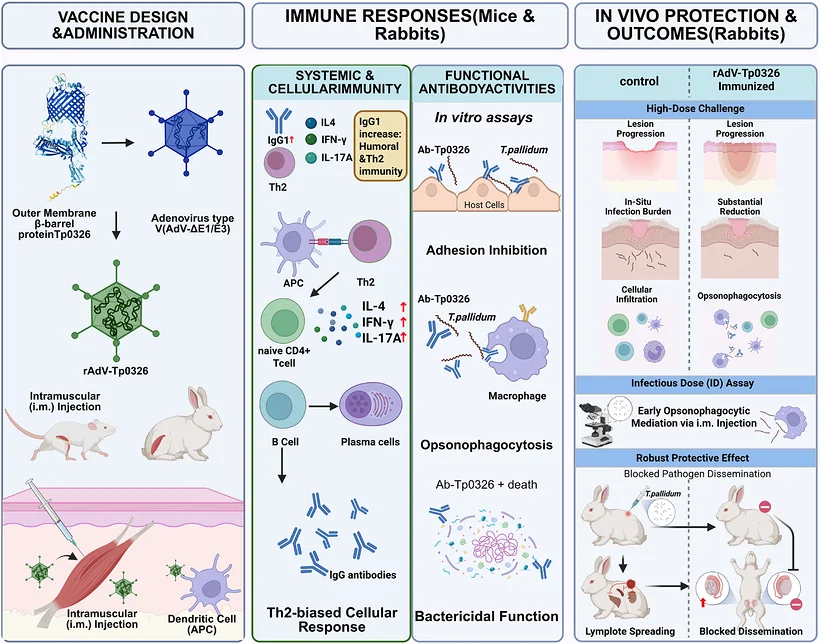

## 1 Introduction

Syphilis, caused by *Treponema pallidum* subspecies *pallidum* (*T. pallidum*), is one of the most prevalent sexually transmitted infections worldwide, with an estimated six million new cases reported annually [1]. *T. pallidum* can be transmitted through sexual contact, blood transfusion, and vertical transmission from mother to child. When syphilis in pregnancy is untreated, treated late, or inadequately treated, 50%–80% of cases result in adverse birth outcomes, including stillbirth, neonatal death, preterm birth, low birth weight, or congenital infection [2]. According to estimates from the World Health Organization (WHO), approximately 700 000 new cases of congenital syphilis occurred globally in 2022, resulting in nearly 390 000 adverse birth outcomes [1, 2]. In China, the elimination of syphilis remains challenging amid a persistently high burden of syphilis in the general population, with 52 197 new cases reported in December 2025 alone and syphilis ranking among the top three most commonly reported Class B notifiable infectious diseases [3, 4]. Congenital syphilis incidence had historically exceeded WHO elimination thresholds (≥50 per 100 000 live births), peaking at 69.9 per 100 000 live births in 2013 before declining to 11.9 per 100 000 live births in 2019 [5]. Similar alarming trends have been observed in other high‐income countries, including Canada, where reported congenital syphilis cases more than doubled between 2016 and 2020 [6]. As the second leading cause of preventable stillbirth globally, congenital syphilis continues to represent a substantial public health burden, with incidence rates remaining more than tenfold above the WHO elimination target [1, 2, 7, 8]. Vaccination, as one of the most effective and cost‐efficient preventive strategies, provides a unique opportunity to confer immune protection before or during pregnancy and has therefore become a critical public health priority for eliminating congenital syphilis and protecting newborns [9, 10, 11].

Several maternal vaccines, including those against rubella [12], hepatitis B virus [13], and varicella [14], have been approved for the prevention of congenital infections, providing a validated paradigm for vaccines targeting vertically transmitted pathogens. Maternal immunization protects the fetus and infant mainly through transplacental transfer of pathogen‐specific IgG antibodies, conferring early‐life passive immunity [15, 16, 17, 18]. Consistent with this concept, maternal‐fetal transmission of syphilis is markedly lower in late infection (∼10%) than in early infection (40%–70%) [19], indicating that adaptive immune responses can effectively reduce vertical transmission and supporting the potential of vaccination to induce similar protection [11]. Clearance of *T. pallidum* is thought to rely largely on antibody‐mediated opsonization [20], but identification of opsonic targets has been hindered by the organism's fragile outer membrane and the scarcity of surface‐exposed transmembrane proteins [21]. In this context, a highly conserved ∼92‐kDa outer membrane protein, TP0326 (BamA), has been identified with opsonophagocytic potential, highlighting it as a promising antigen for antibody‐based vaccine development [22, 23, 24, 25, 26].TP0326 is predicted to be a core component of a hybrid complex integrating β‐barrel assembly and translocation‐assembly modules, thereby contributing to outer membrane biogenesis, a process essential for the survival of *T. pallidum* [21]. Global multicenter sequencing analyzes further demonstrated that sequence variations are predominantly mapped to the extracellular loops (ECLs), particularly ECL4 in TP0326, and structurally related outer membrane β‐barrel proteins map consistently onto predicted three‐dimensional (3D) structural models, indicating a high degree of structural conservation across strains [25, 27, 28]. Using gel microdroplet‐based topological analysis combined with epitope mapping, extracellular loop 4 (ECL4) of TP0326 was identified as an immunodominant region reactive with sera from patients with syphilis [26, 29]. Phage display‐derived monoclonal antibodies targeting ECL4 exhibited robust opsonophagocytic and bacteriostatic activity in vitro and synergized with immune effector cells to mediate pathogen clearance [21]. Consistent with these findings, truncated subunit and DNA vaccines targeting TP0326 conferred partial protective efficacy in a New Zealand rabbit model, supporting TP0326 as a biologically and immunologically plausible candidate antigen for syphilis vaccine development [22, 30].

To translate the immunological potential of TP0326 into a strategy for preventing congenital syphilis, we sought a vaccine platform capable of rapidly establishing maternal protective immunity before or during pregnancy. This goal was driven by the need for not only high levels of antibodies to block vertical transmission but also the rapid induction of potent pathogen‐clearing antibodies to reduce maternal bacteremia and limit placental dissemination. Considering the large molecular weight of TP0326, its low concentration in the extracellular membrane of *T. pallidum*, and the challenges of heterologous expression, we employed complementary antigen presentation methods to compare the full‐length protein with its ECL4 epitope. Therefore, we evaluated three platforms within a unified framework: (i) recombinant full‐length rTP0326 expressed in *Escherichia coli* (*E. coli)*; (ii) TP0326‐ECL4 display based on a *pyrococcus furiosus* thioredoxin (*Pf*Trx) scaffold; and (iii) a replication‐defective type 5 adenovirus vector (rAd5) for efficient in vivo antigen delivery and functional antibody induction. Immunogenicity and protective efficacy were evaluated in mouse and rabbit models using different immunization routes and challenge conditions.

## 2 Result

### 2.1 Preparation and Validation of Recombinant Antigens and Adenoviral Vaccine Vectors

We successfully generated and characterized two key classes of immunogens: recombinant TP0326‐based proteins and a replication‐deficient adenovirus vaccine vector. For protein production, the genes encoding full‐length TP0326 (rTP0326‐6xHis) and its extracellular loop 4 fused to a thioredoxin carrier (r*Pf*Trx‐TP0326^ECL4^‐6xHis) were cloned into pET‐28a(+) vectors and expressed in *E. coli* (Figure 1). Both proteins were purified to >90% homogeneity using nickel‐affinity chromatography, with identity confirmed by Western blot analysis showing bands at the expected molecular weights (Figure S1). The purified proteins were desalted, concentrated, and quantified for use as antigens. In parallel, the recombinant adenovirus Ad5‐TP0326 was constructed to enable eukaryotic expression of TP0326 in vivo. The TP0326 gene was inserted into a shuttle plasmid and homologously recombined with the adenoviral backbone plasmid pAdEasy‐1 in BJ5183 cells (Figure S2a). To validate the successful packaging of the recombinant virus and the expression of the target gene, HEK293T and Sf1ep cottontail rabbit epithelial cells were infected with the viral stock. At 48 h post‐infection, GFP expression was observed (Figure S2b,c), and Western blot analysis of cell lysates using an anti‐Flag antibody confirmed the presence of TP0326 at the expected molecular size in both cell lines (Figure S2d). The recombinant adenovirus was amplified in HEK 293 cells and purified by CsCl density gradient centrifugation. Viral titers were determined to be 1 × 10^10^ VP/mL for rAd5‐TP0326 and 2 × 10^10^ VP/mL for the control virus Ad5(wt). Structural integrity was confirmed by electron microscopy, which revealed the characteristic icosahedral morphology of adenovirus particles (Figure S2e). Together, these efforts yielded well‐characterized protein and viral‐based vaccine candidates, enabling a direct comparison of their immunogenicity and protective efficacy in subsequent animal studies.

**FIGURE 1:**
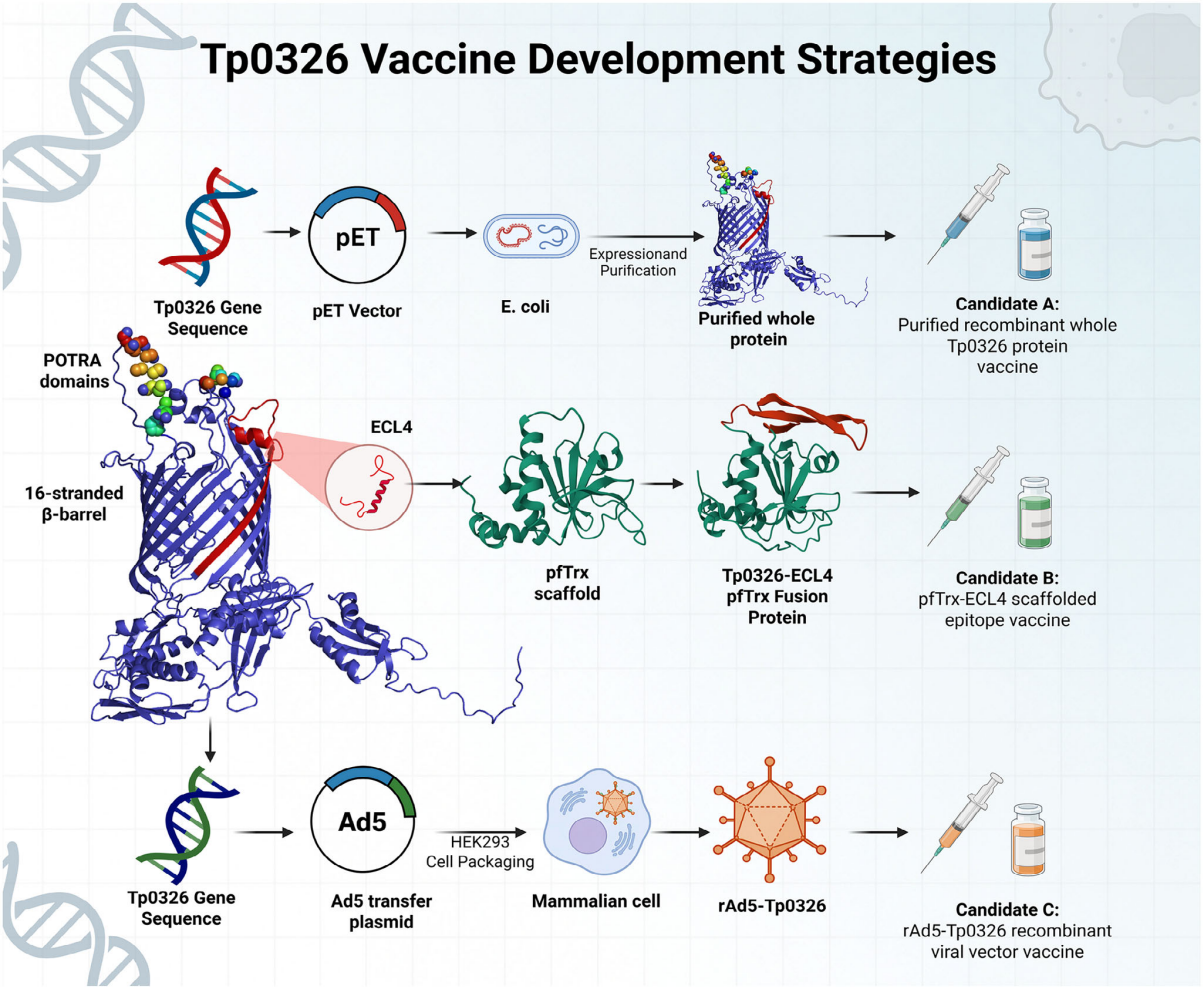
Schematic overview of recombinant protein and adenoviral vector vaccine preparation. Scheme 1 is a schematic diagram of the preparation of recombinant protein vaccines and recombinant adenovirus type 5 vaccines through the Escherichia coli expression system and eukaryotic system respectively. This diagram was created by BioRender. The complete protein sequence was constructed into the prokaryotic expression plasmid pET28a(+) and the adenovirus type 5 defective vector pAd5‐IRES‐GFP respectively, while the extracellular loop 4 was fused with the scaffold protein pfTrx and expressed through the E. coli expression system to form a subunit vaccine. The recombinant protein vaccines and the fusion subunit vaccines were purified through Ni‐NAT resin. The recombinant adenovirus vaccines were obtained after packaging through infection of mammalian cells, and the final animal immunization was used to evaluate their immunogenicity and protective immunity.

### 2.2 Systemic Analysis of Humoral and Cellular Immune Responses of TP0326 Expressed by Replication‐Defective Adenovirus in Mice

To evaluate the immunogenicity of the TP0326 protein in a murine model, BALB/c mice were immunized with either the eukaryotic‐expressed recombinant adenovirus rAd5‐TP0326 or the prokaryotic‐expressed recombinant protein rTP0326. Control groups received r*Pf*Trx‐TP0326^ECL4^, Ad5(wt), or PBS (Figure 2a). Serum antibody titers were determined using recombinant whole Tp0326 protein as the coating antigen and result analysis demonstrated that immunization with rTP0326, r*Pf*Trx‐TP0326^ECL4^, and rAd5‐TP0326 induced high titers of antigen‐specific IgG antibodies. The rTP0326 protein group yielded the highest antibody titers (endpoint titer ≥ 500 000), followed by the r*Pf*Trx‐TP0326^ECL4^ group (≥ 140,000), and the rAd5‐TP0326 group (≥ 50 000). No positive response was detected in the Ad5(wt) or PBS control groups (Figure 2b). Antibody isotyping indicated a Th2‐biased immune response (IgG1/IgG2a >1) in the rTP0326, Ad5(wt), and rAd5‐TP0326 groups (Figure 2c). Subsequent Western blot analysis using post‐immunization mouse sera against varying amounts of rTP0326 and *T. pallidum* lysates revealed that, despite lower anti‐rTP0326 antibody titers, sera from the rAd5‐TP0326 group exhibited the strongest reactivity with *T. pallidum* lysates. In contrast, sera from the rTP0326 and r*Pf*Trx‐TP0326^ECL4^ groups showed weaker reactivity, while the Ad5(wt) group showed no reaction with either rTP0326 or the *T. pallidum* lysates (Figure 2d). This suggests that eukaryotic expression facilitates the formation of a protein conformation closer to the native state, thereby eliciting antibodies targeting natural epitopes. Functional assays were conducted to evaluate the protective capacity of the induced antibodies. After co‐incubation of *T. pallidum* with post‐immunization sera from each group, the mixture was added to Sf1ep cell cultures. After 48 h, the adhesion rate was calculated as the ratio of *T. pallidum* counts in the supernatant to the initial inoculum using a darkfield microscope (Figure 2e) and adhesion was visualized via cell immunofluorescence (Figure 2f). The results demonstrated potent Fc receptor‐independent activities. Sera from both the rTP0326 and rAd5‐TP0326 groups significantly inhibited the adhesion of T. pallidum to Sf1Ep cells at the 48 h time point (Figure 2e), whereas after 7 days of co‐culture, these sera effectively suppressed the viability and growth of the pathogen in vitro (Figure 2g). Furthermore, when *T. pallidum* was opsonized with mouse sera and then co‐cultured with murine bone marrow–derived macrophages, sera from the r*Pf*Trx‐TP0326^ECL4^, rTP0326, and rAd5‐TP0326 groups all promoted significant opsonophagocytosis of both the Nichols, smudh02_SS14 and smudh04_SS14 strains, an Fc receptor‐dependent function (Figure 2h and Figure S3). Collectively, these findings indicate that while the rAd5‐TP0326 vaccine induced lower absolute antibody titers than the protein vaccines, it generated high‐quality functional antibodies with the potential to neutralize and clear the pathogen. To assess T‐cell‐mediated immunity, splenocytes were isolated and stimulated in vitro post‐immunization. Flow cytometric analysis of splenocytes revealed no marked T‐cell response in the rAd5‐TP0326 group (Figure S4a–d). However, EdU proliferation assays showed significant antigen‐specific proliferation of T lymphocytes from mice immunized with rTP0326 or rAd5‐TP0326 (Figure S4e). Cytokine profiling of rTP0326‐stimulated splenocytes by ELISA revealed a significant increase only in interleukin‐17a (IL‐17a) levels, whereas typical Th1 cytokines such as IL‐2, IFN‐γ, and TNF‐α showed no significant difference compared to controls (Figure S4f). This indicates that the candidate vaccines did not elicit an obvious Th1‐type cellular immune response.

**FIGURE 2:**
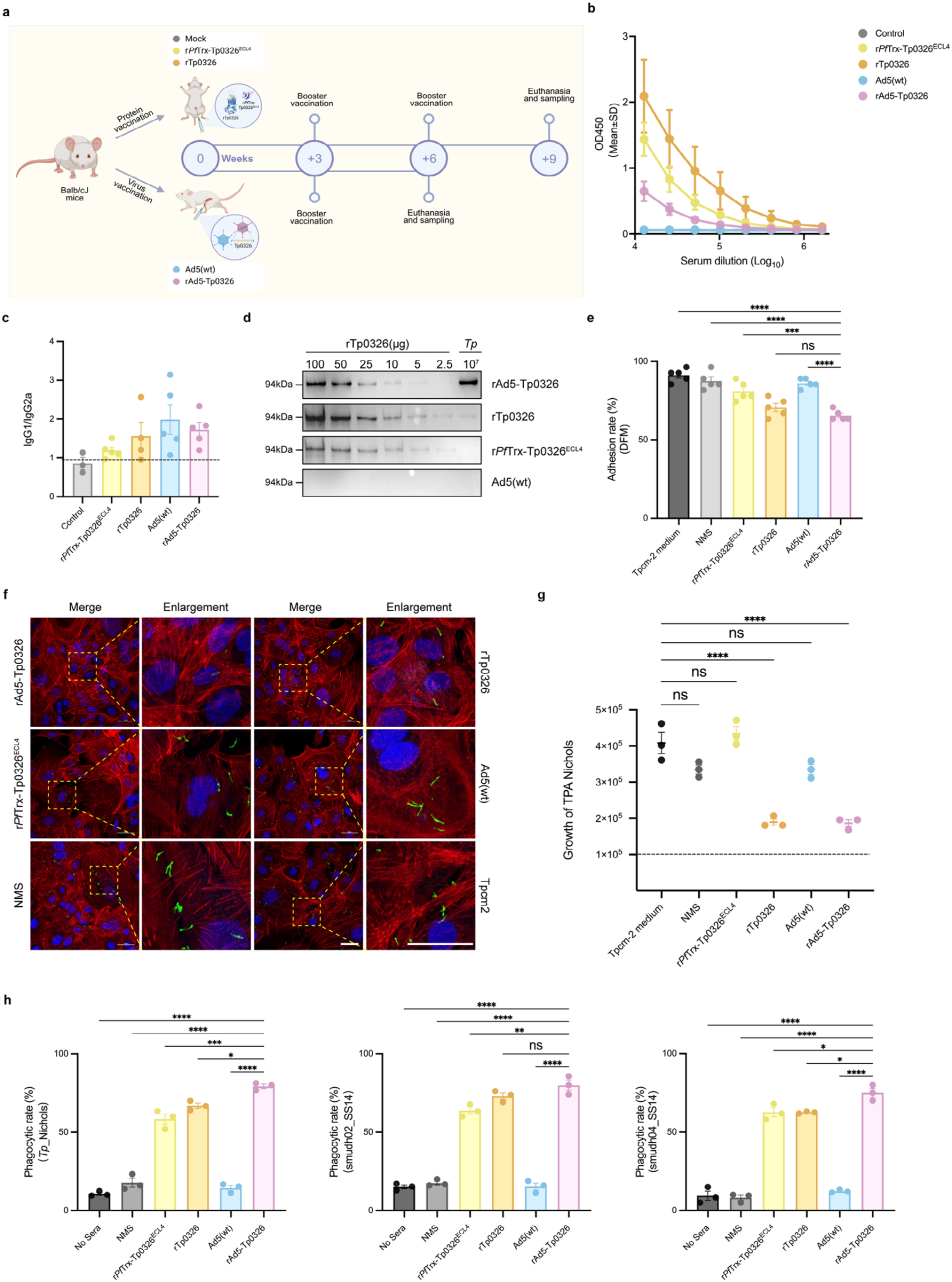
The immunological activity of serum antibodies in mice immunized with recombinant adenovirus and protein vaccines. (a) Flow chart of immunization plan for Balb/c Mice (*n* = 5, respectively). (b) Detection of specific antibody titers at the immune endpoint in the serum of mice immunized with different vaccine groups. (c) The ratio of serum antibody IgG1 to IgG2a in each vaccine immunization group. (d) The antigen sensitivity of antibodies in each vaccine group and the detection of *T.pallidum* lysat. (e) Detection of the effects of antisera from each group on the adhesion of *T.pallidum* to cells by dark‐field microscopy. (f) Detection of the effects of different sera on the adhesion of *T.pallidum* to cells by indirect immunofluorescence. (g) Bactercidal of antibodies from different groups on the growth of *T.pallidum* cultivation in vitro. (h) The phagocytosis rate of each group was statistically analyzed according to the number of *T.pallidum* phagocytized by macrophage and separate tests were conducted on the Nichols reference strain and clinical strains. n.s.: no significance.

### 2.3 Evaluation of the Immunogenicity of TP0326 Expressed by Replication‐defective adenovirus in New Zealand Rabbits

The immunogenicity of the TP0326 vaccines was further validated in a New Zealand rabbit model, with a focus on the humoral immune response (Figure 3a). Consistent with the murine data, the rTP0326 protein group induced the highest antibody titers in rabbits (endpoint titer ≥ 140 000), followed by the r*Pf*Trx‐TP0326^ECL4^ group (≥80 000) and the rAd5‐TP0326 group (≥ 20 000). The Ad5(wt) and PBS control groups remained negative (Figure 3b). Serum samples were collected from immunized rabbits prior to challenge to evaluate the systemic cytokine responses elicited by the different vaccine regimens. Analysis of serum cytokine levels by ELISA revealed that Interferon‐gamma(IFN‐γ) levels were significantly elevated in all immunized groups compared to the control group (*p* < 0.05), indicating a broad induction of a Th1‐type immune response following vaccination. Similarly, interleukin‐4 (IL‐4) levels showed increases in all vaccinated groups relative to controls (Figure 3c). However, statistical significance for IL‐4 elevation was achieved only in the r*Pf*Trx‐TP0326^ECL4^ group and the rAd5‐TP0326 group (*p* < 0.05), suggesting a more pronounced Th2‐type response in these particular groups. In contrast, no significant differences in interleukin‐17a (IL‐17a) levels were observed among any of the groups. Western blot analysis again confirmed that rabbit sera from the rAd5‐TP0326 group exhibited superior binding to native *T. pallidum* proteins, reinforcing the advantage of the eukaryotic expression system in preserving native antigen conformation (Figure 3d). In functional assays, the rAd5‐TP0326 immune sera demonstrated a strong capacity to inhibit treponemal adhesion to host cells, second only to sera from infected rabbits (IRS) (Figure 3e,f). Additionally, IRS and sera from the rTP0326, r*Pf*Trx‐TP0326^ECL4^ and rAd5‐TP0326 groups effectively suppressed the growth of *T. pallidum* in vitro (Figure 3g). Notably, the bactericidal effect observed in the rAd5‐TP0326 group was superior to that achieved with IRS. Opsonophagocytosis assays confirmed that IRS and antibodies induced by rTP0326, r*Pf*Trx‐TP0326^ECL4^, and rAd5‐TP0326 groups significantly enhanced the phagocytosis of both Nichols and SS14 strains by macrophages (Figure 3h and Figure S5).

**FIGURE 3:**
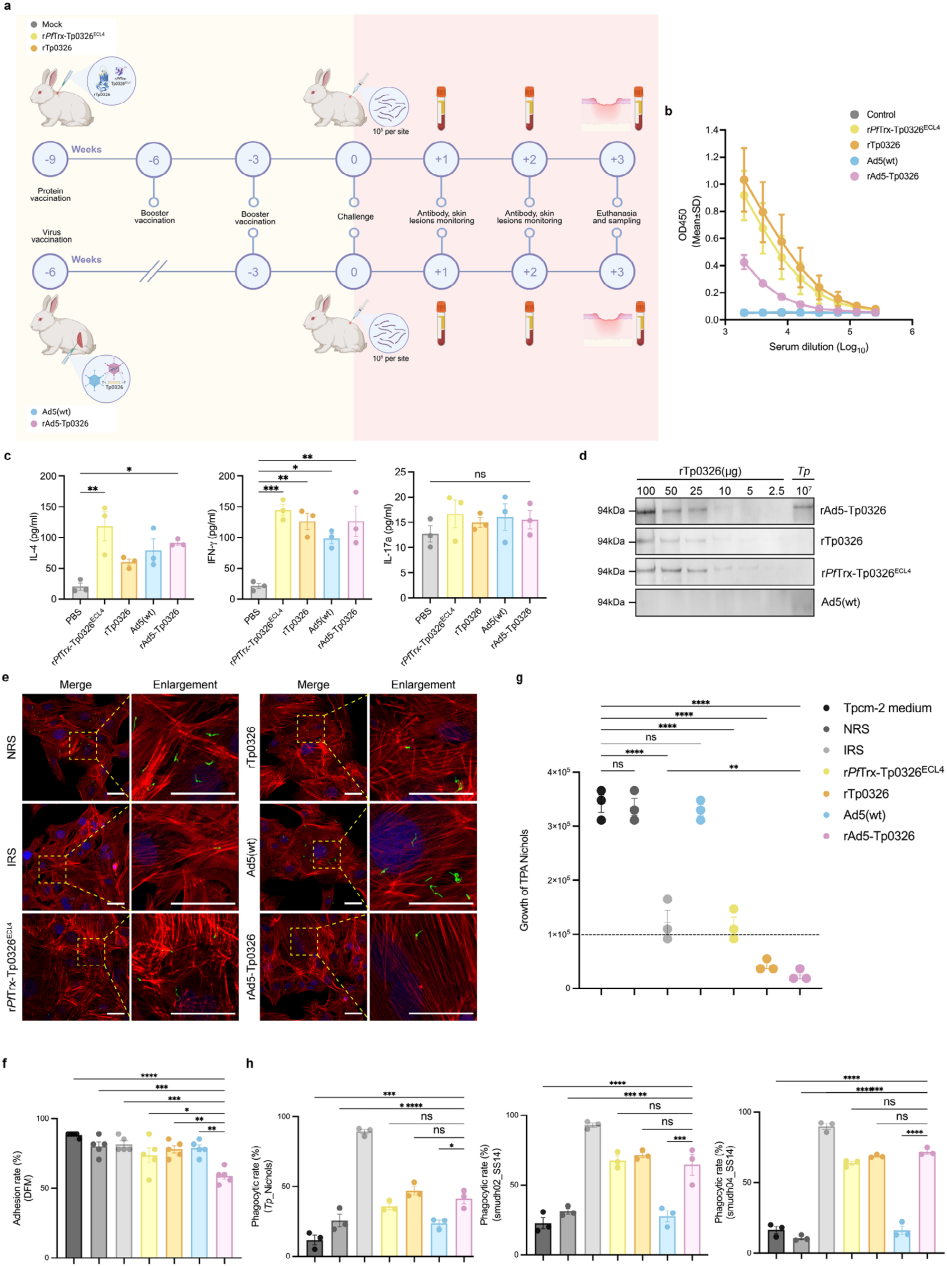
The immunological activity of antibodies in rabbit Sera immunized with recombinant adenovirus and protein vaccines. (a) Flow chart of immunization and challenge plan for New Zealand rabbits (*n* = 3, respectively). (b) Detection of specific antibody titers at the immune endpoint in the serum of rabbits immunized with different vaccine groups. (c) The concentrations of interleukin‐4 (IL‐4), interleukin‐17A (IL‐17A), and interferon‐gamma (IFN‐γ) in the sera of each vaccine immunization group. (d) The antigen sensitivity of antibodies in each vaccine group and the detection of *T.pallidum* lysate. (e) Detection of the effects of different sera on the adhesion of *T.pallidum* to cells by indirect immunofluorescence. (f) Detection of the effects of antisera from each group on the adhesion of *T.pallidum* to cells by dark‐field microscopy. (g) Bactercidal of antibodies from different groups on the growth of *T.pallidum* cultivation in vitro. (h) The phagocytosis rate of each group was statistically analyzed according to the number of *T.pallidum* phagocytized by macrophage and separate tests were conducted on the Nichols reference strain and clinical strains. n.s.: no significance.

### 2.4 rAd5‐TP0326 Immunization Confers Partial Protection Against High‐Dose *T. Pallidum* Challenge

To assess the protective efficacy of the rAd5‐TP0326 vaccine candidate, rabbits were immunized according to specific regimens. The rTP0326 and r*Pf*Trx‐TP0326^ECL4^ groups received antigens emulsified first in Freund's adjuvant, followed by two boost vaccinations with a non‐Freund's adjuvant at three‐week intervals. In contrast, the Ad5(wt) and rAd5‐TP0326 groups were administered two intramuscular immunizations at three‐week intervals. Control studies using an adjuvant‐only regimen confirmed the absence of protective immunity from the adjuvant alone, ruling out its contribution to the observed effects. Three weeks after the final immunization, both immunized and control animals were challenged intradermally with 1 × 10^5^ *T. pallidum* subsp. *pallidum*, Nichols strain, at each of eight sites on their shaved backs. Lesion development was monitored over the following 21 days (Figure 4a). By day 21 post‐challenge (PC), primary lesions had developed at all inoculation sites on the backs of all rabbits in every group. Although the r*Pf*Trx‐TP0326^ECL4^ group exhibited a trend toward attenuated ulcer progression, with a reduced ulceration rate of 54.1% and a smaller mean lesion diameter of 13.96 ± 0.35 mm, these values did not differ significantly from those of the unimmunized control group, which displayed 100% ulceration and a larger mean lesion diameter of 15.75 ± 0.52 mm (*p* > 0.05). The rTP0326 group also demonstrated reduced ulceration rates (50%, *P*> 0.05) and lesion diameters (11.54 ± 0.79 mm, *P* <0.05) relative to controls. Notably, the Ad5(wt) group displayed attenuated lesion progression, with ulceration rates (33.3%, *P*> 0.05) and lesion diameters (10.67 ± 0.89 mm, *P* <0.05) lower than the control group, second only to the rAd5‐TP0326 group. The rAd5‐TP0326 immunized group exhibited a complete absence of ulceration (0%, *P* <0.05) and the smallest mean lesion diameter (8.5 ± 0.52 mm, *P* <0.05) among all groups (Table 1). Serological analyses performed every 7 days revealed no statistically significant differences in TPPA titers among the groups (Figure 4b). However, RPR titers were lower in all immunized groups compared to the unimmunized control group, with the rAd5‐TP0326 group showing the lowest reactivity (Figure 4b). On day 21 post‐challenge (PC), animals were euthanized, and samples from skin lesions, spleen, and liver were collected. Darkfield microscopy analysis of lesion exudates confirmed the presence of motile *T. pallidum* in all groups (Figure S7). The proportion of darkfield‐positive lesions was 55.5% (*P*> 0.05) in both the rTP0326 and rAd5‐TP0326 groups, which was lower than in other groups but not statistically significant (Figure 4d). Quantitative PCR (qPCR) targeting the *polA* gene, normalized to the host β‐actin gene, was used to assess bacterial burden. All immunized groups had significantly lower *T. pallidum* loads in skin lesions compared to the control group, with the rAd5‐TP0326 group showing the most substantial reduction (Figure 4c). In the spleen, all immunized groups also exhibited reduced bacterial burdens compared to controls, with the rTP0326 group displaying the lowest load. Due to low normalized *polA* copy numbers, no significant differences were detected in the bacterial loads in the liver or blood between groups (Figure 4c). Collectively, these results indicate that immunization with rAd5‐TP0326 attenuates lesion development and to a certain extent, it can also inhibits the dissemination of *T. pallidum* to distant organ sites.

**FIGURE 4:**
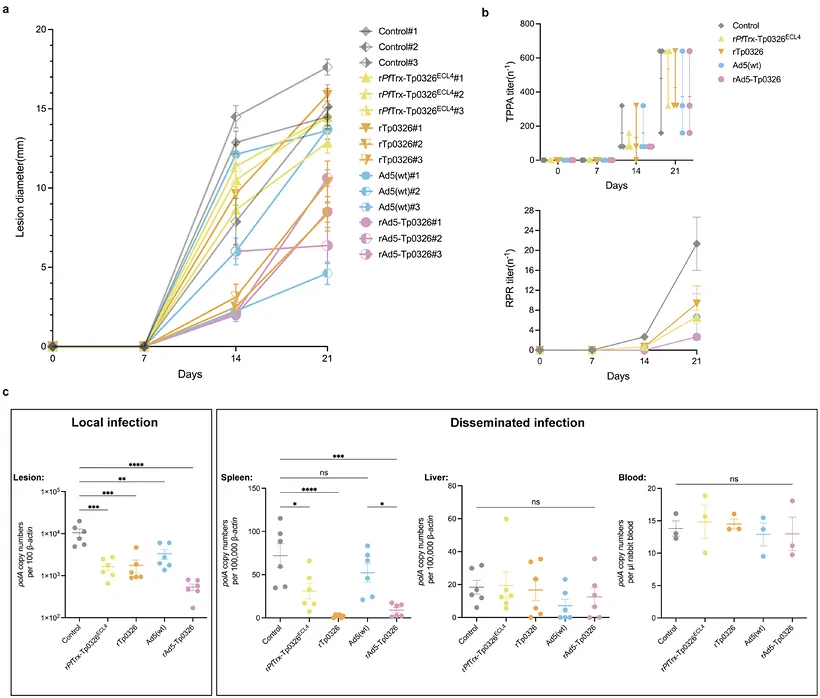
Immune protection of rabbits immunized with different vaccines against *T.pallidum* Challenge. (a) Changes in the diameter of chancres following intradermal *T.pallidum* challenge in rabbits (X = 8/rabbit, respectively). (b) Specific and non‐specific antibodies induced by T.pallidum infection were detected using the *T.pallidum* particle agglutination assay (TPPA) and the Rapid Plasma Reagin (RPR) test, respectively. (c) The polA gene was amplified by quantitative polymerase chain reaction (qPCR) to measure the *T.pallidum* burden in the skin at the local site, distal organs including the spleen and liver, and in the blood. n.s.: no significance.

**TABLE 1:** Effects of different Tp0326‐Based vaccines on lesion progression at Day 21 Post‐Challenge.

Groups	Wheals or papules/inoculation sites(%)	Ulcers/inoculation sites (%)	*p* value	Lesion diameter (mm)(± SEM)	*p* value	Lesions positive for *T. pallidum* (%)	*p* value
Control	24/24(100)	24/24(100)	—	15.75 ± 0.52	—	9/9(100)	—
r*Pf*Trx‐Tp0326^ECL4^	24/24(100)	13/24(54.1)	>0.05	13.96 ± 0.35	>0.05	8/9(88.8)	>0.05
rTp0326	24/24(100)	12/24(50)	>0.05	11.54 ± 0.79	<0.05	5/9(55.5)	>0.05
Ad5(wt)	24/24(100)	8/24(33.3)	>0.05	10.67 ± 0.89	<0.05	8/9(88.8)	>0.05
rAd5‐Tp0326	24/24(100)	0/24(0)	<0.05	8.50 ± 0.52	<0.05	5/9(55.5)	>0.05

### 2.5 rAd5‐TP0326 Immunization Mitigates Histopathological Damage and is Associated With Opsonophagocytosis In Vivo

Histopathological examination of lesion biopsies collected on day 21 PC revealed severe tissue damage in unimmunized control animals, characterized by a loss of intact epidermal architecture. The r*Pf*Trx‐TP0326^ECL4^ and Ad5(wt) groups displayed milder pathology, while the least severe damage was observed in the rTP0326 and rAd5‐TP0326 groups (Figure 5a). Immunohistochemical staining for CD68 showed no significant differences in macrophage infiltration among the groups (Figure 5b). Immunofluorescence staining for *T. pallidum* revealed distinct patterns: control and Ad5(wt) groups exhibited typical spiral fluorescence corresponding to intact spirochetes, whereas the r*Pf*Trx‐TP0326^ECL4^, rTP0326, and rAd5‐TP0326 groups showed punctate fluorescence patterns (Figure 5c). The presence of punctate fluorescent signals, interpreted as internalized treponemal debris, provides histological evidence consistent with our in vitro opsonophagocytosis data. This correlation suggests that the promoted phagocytic activity is not merely an in vitro phenomenon but is functionally operative in vivo, likely contributing to the containment of the infection at the local site.

**FIGURE 5:**
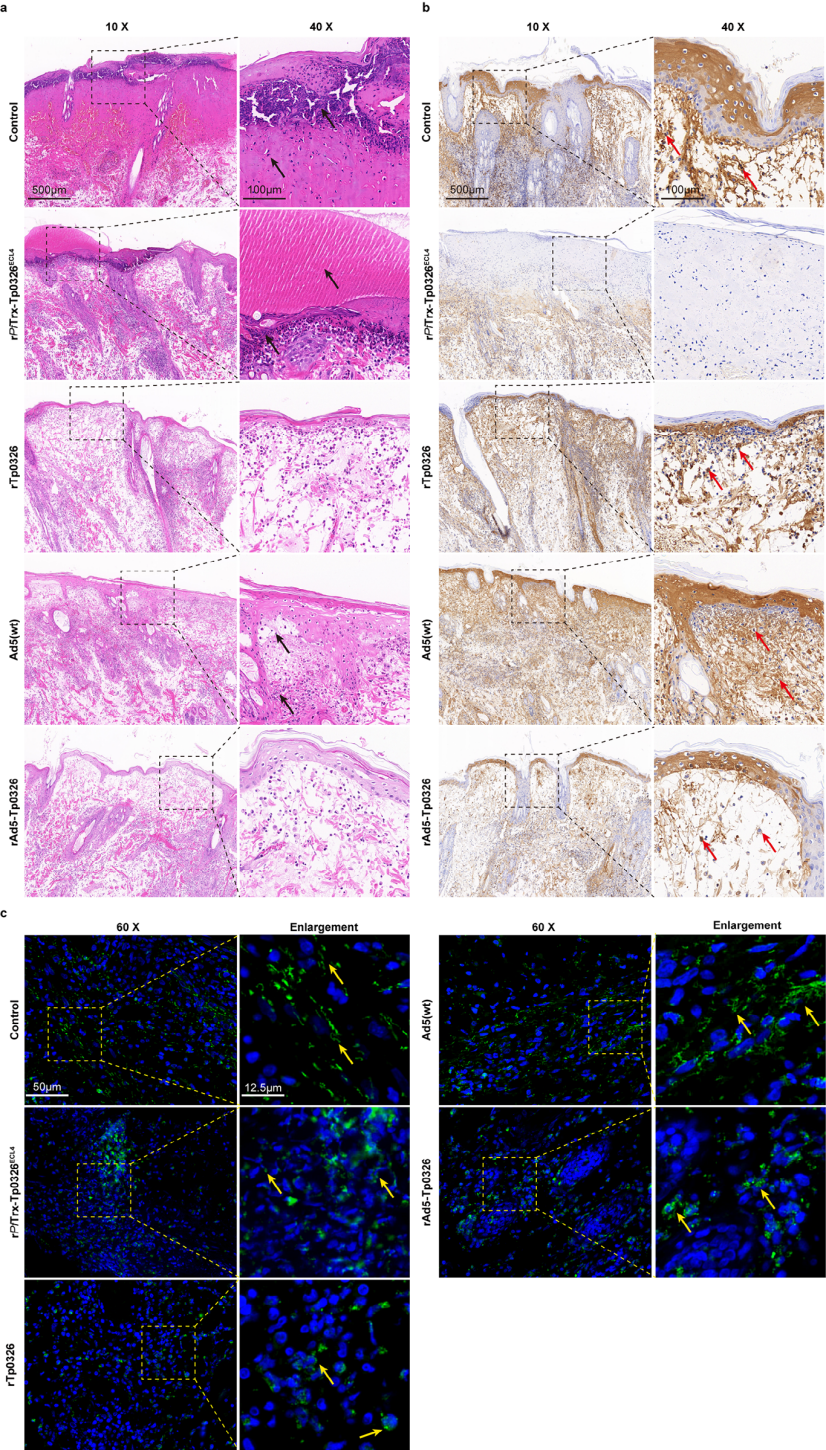
Histopathological of the skin in vaccinated rabbits post‐21 days intradermal *T.pallidum* challenge. (a) Representative hematoxylin and eosin (H&E)–stained sections of the epidermis from rabbits in each group. Black arrows indicate representative areas of tissue injury, including cellular necrosis, inflammatory cell infiltration, and disruption of tissue architecture. (b) Representative immuno‐histochemical staining for CD68 in the epidermis from rabbits in each group. Red arrows indicate CD68‐positive macrophages. (c) Representative immunofluorescence staining of *T. pallidum* in the epidermis from rabbits in each group. Yellow arrows indicate different green fluorescent signal patterns of *T. pallidum* observed under microscopy. H&E and CD68‐stained images were captured at ×100 magnification (scale bar = 500 µm) and further examined at ×400 magnification (scale bar = 100 µm).*T. pallidum* immunofluorescence images were acquired at ×600 magnification (scale bar = 50 µm) and digitally zoomed by 4× (effective scale bar = 12.5 µm).

### 2.6 Superior Protective Efficacy of rAd5‐TP0326 Vaccine Against Infective Dose *T. Pallidum* Challenge

Building upon the results from the high‐dose challenge, which demonstrated that the rAd5‐TP0326 vaccine conferred the most significant protection against lesion development and bacterial dissemination, we sought to further evaluate its efficacy under conditions that more closely mimic natural infection. To this end, a subsequent experiment was conducted using a lower challenge dose of 1×10^2^ *T. pallidum* per site. Furthermore, to explore the impact of immunization route on protective efficacy, we compared the performance of the rAd5‐TP0326 vaccine administered via two distinct pathways: intramuscular (i.m.) and intranasal (i.n.) (Figure 6a). This refined experimental design allowed us to rigorously assess whether the superior protection observed with the rAd5‐TP0326 vaccine was maintained under a lower infectious burden and to determine the potential influence of the immunization route on the outcome. Pre‐challenge Serological analysis revealed high anti‐rTP0326 antibody titers in both the rAd5‐TP0326 (intramuscular, i.m.) and rAd5‐TP0326 (intranasal, i.n.) groups prior to challenge, with rAd5‐TP0326 (i.n.) group exhibiting the highest level. In contrast, no specific antibodies were detected in the Ad5(wt) control group (Figure 6b). Cytokine profiling by ELISA revealed elevated IFN‐γ levels in the rAd5‐TP0326 (i.n.), Ad5(wt), and rAd5‐TP0326 (i.m.) groups, and increased IL‐4 levels in the rAd5‐TP0326 (i.n.) and rAd5‐TP0326 (i.m.) groups, suggesting the elicitation of a mixed Th1/Th2 immune response (Figure 6c). At day 21 PC, despite the rAd5‐TP0326 (i.n.) group exhibiting the highest antibody titers, the rAd5‐TP0326 (i.m.) group demonstrated superior containment of lesion development, showing the smallest mean lesion diameter (4.29 ± 0.62 mm). This was significantly reduced compared to the control group (12.48 ± 0.39 mm; p < 0.05), the rAd5‐TP0326 (i.n.) group (6.75 ± 0.32 mm), and the Ad5(wt) group (9.88 ± 0.34 mm) (Figure 6d and Table 2). Particularly noteworthy was the complete protection observed in rabbit rAd5‐TP0326 (i.m.)#1, which developed no visible erythema or papules (Figure S9). Ulceration rates remained at 0% in both rAd5‐TP0326 immunization groups, contrasting with the 8.3% rate observed in control and Ad5(wt) groups, though this difference did not reach statistical significance (*p* > 0.05) (Table 2). The lesion development through day 28 PC revealed maintained differential protection among groups. Ulceration rates substantially increased to 75% in both control and Ad5(wt) groups, while the rAd5‐TP0326 (i.n.) and rAd5‐TP0326 (i.m.) groups showed markedly lower rates of 33.3% and 20.8%, respectively. All three immunized groups continued to exhibit significantly smaller lesion diameters compared to the control group, with the hierarchical protection pattern remaining consistent. By the study endpoint at day 35 PC, the rAd5‐TP0326 (i.n.) group displayed the highest ulceration rate (95.8%), whereas the rAd5‐TP0326 (i.m.) group maintained the most favorable profile with the lowest rate (50%). A remarkable reversal in lesion dynamics was observed: while control, Ad5(wt), and rAd5‐TP0326 (i.m.) groups showed beginning resolution of lesion diameters, the rAd5‐TP0326 (i.n.) group demonstrated continued progression, reaching the largest mean diameter (14.38 ± 0.69 mm) which exceeded both control (11.35 ± 0.78 mm) and Ad5(wt) (13.58 ± 0.61 mm) groups. Throughout the observation period, the rAd5‐TP0326 (i.m.) group consistently maintained the smallest lesion diameter (8.58 ± 1.30 mm) (Table S3). Quantitative PCR (qPCR) analysis of DNA extracted from lesions was performed to assess the local bacterial burden at the site of infection. The results demonstrated a significant reduction in *T. pallidum* loads across all immunized groups compared to the unimmunized control group (Figure 6e). Notably, the lowest bacterial loads were confirmed in the rAd5‐TP0326 (i.n.) and rAd5‐TP0326 (i.m.) groups, consistent with their observed delay in lesion progression. Darkfield microscopy revealed a lower rate of lesions positive for *T. pallidum* in the rAd5‐TP0326 (i.m.) group (33.3%) compared to rAd5‐TP0326 (i.n.) group (66.7%), Ad5(wt) group (66.7%), and the control group (66.7%), although this did not reach statistical significance (*p* > 0.05) (Table 2). Serologically, all immunized groups showed a delayed increase in TPPA titers compared to controls. Notably, in the rAd5‐TP0326 (i.m.) group, rAd5‐TP0326 (i.m.) #1 displayed TPPA titers of 1:40 on days 14 and 21, which declined to 1:20 by day 28 and became seronegative by day 35. In contrast, TPPA titers in the other immunization groups continued to increase over time, albeit remaining lower than those in the control group. At day 21 PC, all rabbits in the control group showed RPR seroconversion. In immunization groups, one rabbit in the Ad5(wt) control group showed RPR seroconversion, while all animals in both rAd5‐TP0326 (i.n.) and rAd5‐TP0326 (i.m.) groups remained RPR‐negative. By day 28 PC, complete seroconversion was observed in the Ad5(wt) control group with rapidly rising RPR titers, and all rabbits in the rAd5‐TP0326 (i.n.) group also became RPR‐positive. rAd5‐TP0326 (i.m.) #1 is the single rabbit which maintained RPR‐negative status, while its two littermates seroconverted with titers stabilizing at 1:8. This pattern continued through day 35 PC, with rAd5‐TP0326 (i.m.) #1 in the rAd5‐TP0326 (i.m.) group maintaining RPR negativity, while the other two rabbits in this group showed stable titers of 1:8. Both the rAd5‐TP0326 (i.n.) and Ad5(wt) control groups exhibited progressively increasing RPR titers, though the control group began to show declining RPR titers by day 35 PC, consistent with the progression of cutaneous lesions (Figure 6f).

**FIGURE 6:**
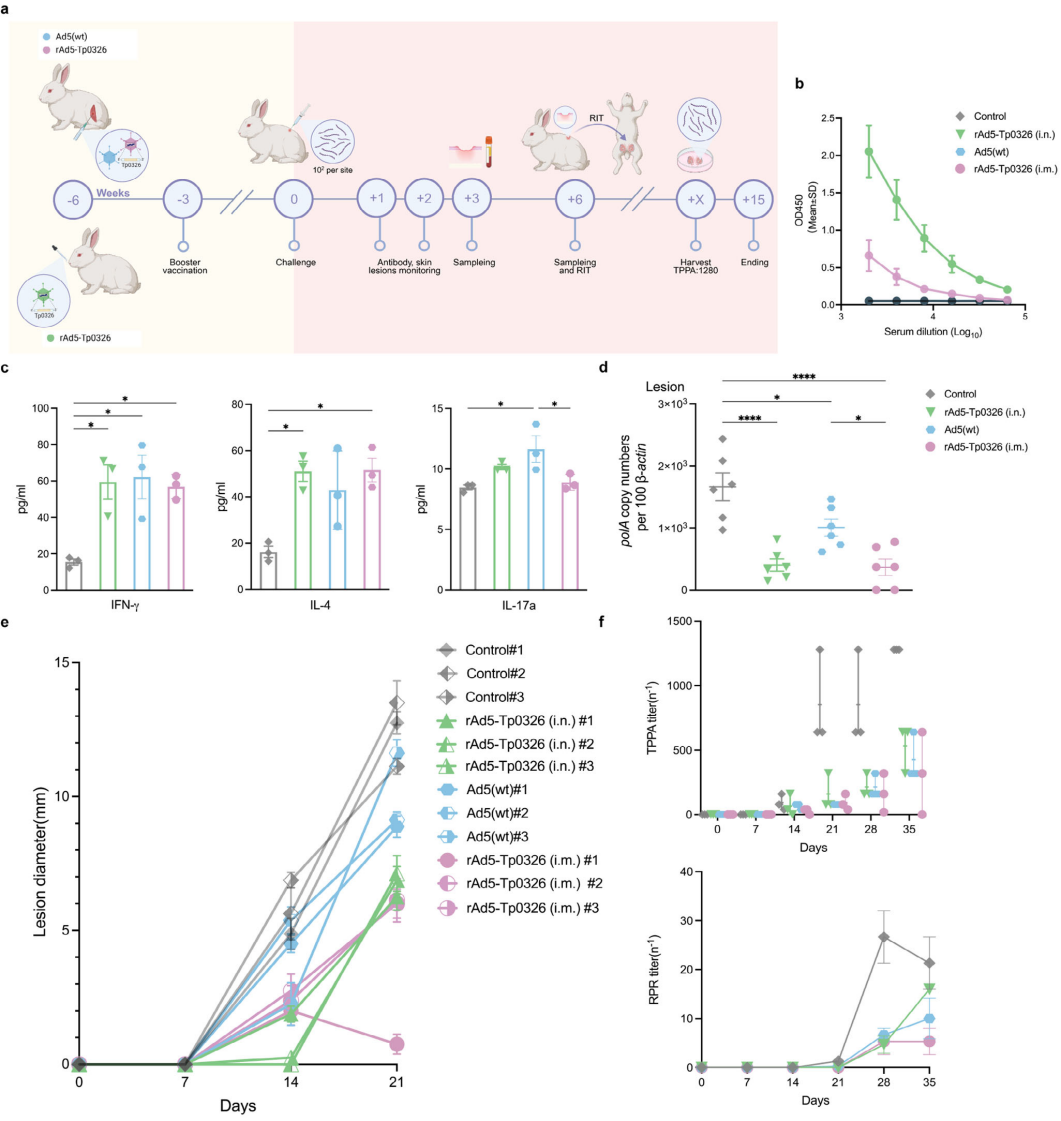
Protective immune responses in rabbits immunized via different routes with a recombinant adenovirus vaccine against *T.pallidum* following a low‐dose challenge (1 × 10^2^ T. pallidum per site). (a) Flow chart of immunization, challenge, and RIT plan for New Zealand rabbits (*n* = 3, respectively). (b) Detection of specific antibody titers at the immune endpoint in the serum of rabbits immunized with different vaccine groups. (c) The concentrations of interleukin‐4 (IL‐4), interleukin‐17A (IL‐17A), and interferon‐gamma (IFN‐γ) in the sera of each vaccine immunization group. (d) Changes in the diameter of chancres following intradermal *T.pallidum* challenge in rabbits (X = 8/rabbit, respectively). (e) Specific and non‐specific antibodies induced by *T.pallidum* infection were detected using the *T.pallidum* particle agglutination assay (TPPA) and the Rapid Plasma Reagin (RPR) test, respectively. (f) The polA gene was amplified by quantitative polymerase chain reaction (qPCR) to measure the *T.pallidum* burden in the skin at the local site. n.s.: no significance.

**TABLE 2:** Effects of different immunization routes of recombinant adenoviral vaccines on lesion progression at day 21 following challenge with a 1 × 10^2^ Infectious Dose.

Groups	Wheals or papules/Inoculation sites (%)	*p* value	Ulcers/Inoculation sites (%)	*p* value	Lesion diameter (mm)(± SEM)	*p* value	Lesions positive for T. pallidum (%)	*p* value
Control	24/24(100)	—	2/24(8.3)	—	12.48 ± 0.39	—	9/9(100)	—
rAd5‐Tp0326(i.n.)	24/24(100)	>0.05	0/24(0)	>0.05	6.75 ± 0.32	<0.05	6/9(66.7)	>0.05
Ad5(wt)	24/24(100)	>0.05	2/24(8.3)	>0.05	9.88 ± 0.34	<0.05	6/9(66.7)	>0.05
rAd5‐Tp0326(i.m.)	16/24(66.6)	>0.05	0/24(0)	>0.05	4.29 ± 0.62	<0.05	3/9(33.3)	>0.05

### 2.7 Evidence of in Vivo Phagocytosis in rAd5‐TP0326 Immunized Rabbits

Histopathological analysis of lesions from the low‐dose challenge revealed generally less severe damage across all groups compared to the high‐dose challenge, with control animals still exhibiting the most significant pathology (Figure 7a). CD68 staining showed no marked differences in macrophage infiltration among groups (Figure 7b). Immunofluorescence analysis revealed linear fluorescence patterns indicative of intact treponemes in the control, Ad5(wt), and rAd5‐TP0326 (i.n.) groups. In contrast, lesions from the rAd5‐TP0326 (i.m.) group showed minimal fluorescence, consistent with the low bacterial burden detected by qPCR. (Figure 7c). Dark‐field microscopic examination of lesion exudates provided critical insights into the status of infection at the local sites. Motile *T. pallidum* was readily observed in exudates from every rabbit in the control, Ad5(wt), and rAd5‐TP0326 (i.n.) groups, indicating active infection at the challenge sites. In stark contrast, examination of lesion exudates from the rAd5‐TP0326 (i.m.) group revealed a complete absence of motile *T. pallidum* in rabbit rAd5‐TP0326 (i.m.) #1, which correlated with the fact that this animal never developed ulceration throughout the study. A significant and novel finding was observed in samples from other rabbits within the rAd5‐TP0326 (i.m.) group that developed lesions. These samples showed clear evidence of *T. pallidum* being internalized by host cells, a phenomenon highly suggestive of active in vivo opsonophagocytosis (Figure 7d and Video S1). To our knowledge, this represents the first direct microscopic observation of such a cellular clearance process in the context of *T. pallidum* infection.

**FIGURE 7:**
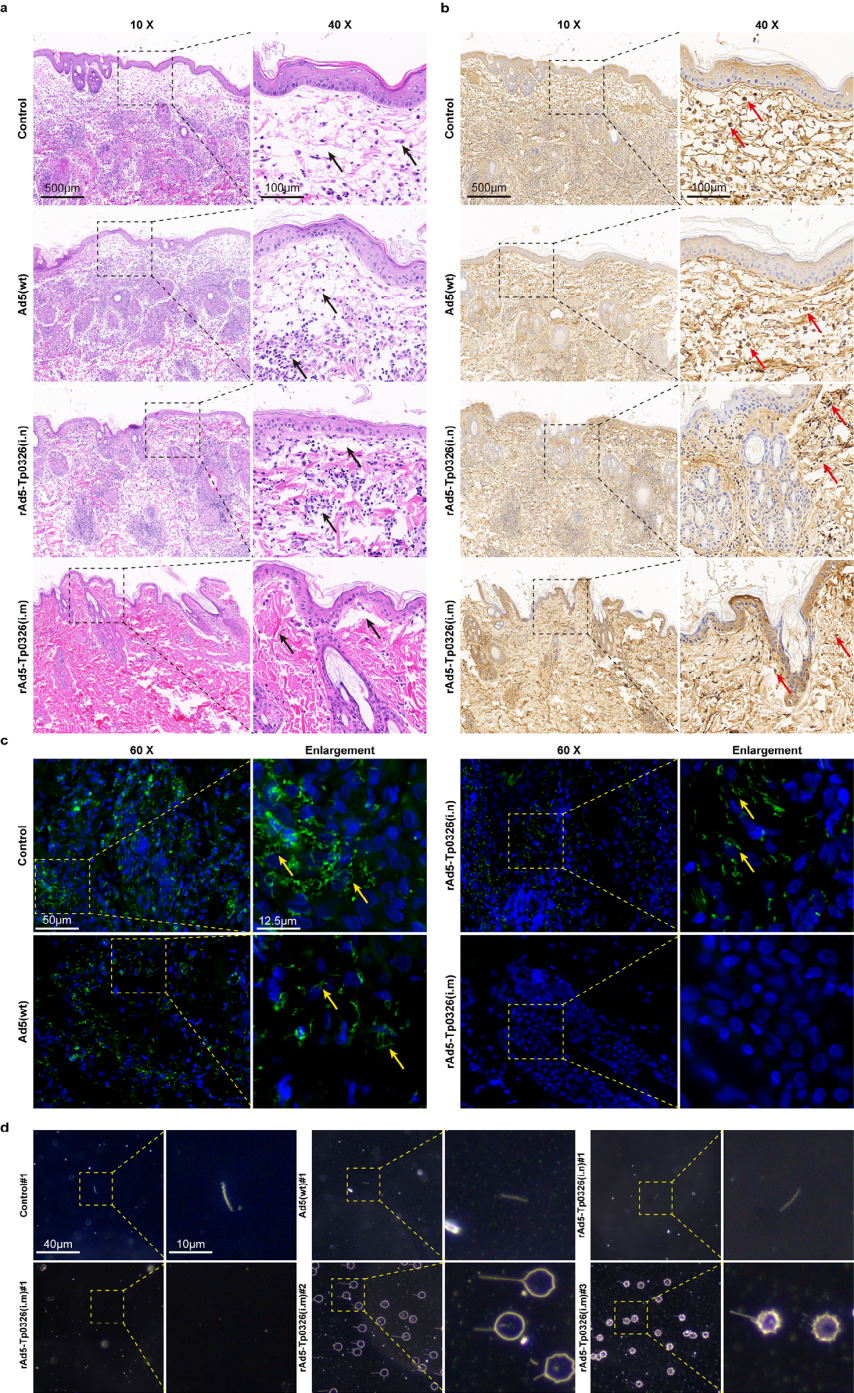
Histopathological of the skin in vaccinated rabbits post‐21 days intradermal *T.pallidum* challenge. (a) Representative hematoxylin and eosin (H&E)‐stained sections of the epidermis from rabbits in each group. Black arrows indicate representative areas of tissue injury, including cellular necrosis, inflammatory cell infiltration, and disruption of tissue architecture. (b) Representative immuno‐histochemical staining for CD68 in the epidermis from rabbits in each group. Red arrows indicate CD68‐positive macrophages. (c) Representative immunofluorescence staining of *T.pallidum* in the epidermis from rabbits in each group. Yellow arrows indicate different green fluorescent signal patterns of *T. pallidum* observed under microscopy. (d) Representative dark‐field microscopic examination of *T. pallidum* in exudates from skin lesions of rabbits in each group. H&E‐ and CD68‐stained images were captured at ×100 magnification (scale bar = 500 µm) and further examined at ×400 magnification (scale bar = 100 µm). *T. pallidum* immunofluorescence images were acquired at ×600 magnification (scale bar = 50 µm) and digitally zoomed by 4× (effective scale bar = 12.5 µm).

### 2.8 rAd5‐TP0326 Immunization Impairs *T. Pallidum* Transmission Following Lesion Transplantation

To further determine whether immunization with rAd5‐TP0326 is capable of preventing the dissemination of *T. pallidum*, lesion tissues were collected from two New Zealand rabbits in each of the following groups: the control, rAd5‐TP0326 (i.n.), Ad5(wt), and rAd5‐TP0326 (i.m.). The harvested lesions were homogenized and individually transplanted into healthy recipient rabbits (Figure 6a). Infection status of the recipient rabbits was evaluated by serological testing using the *T. pallidum* particle agglutination assay (TPPA) and by quantification of *T. pallidum* havested from the testes using dark‐field microscopy, with strong seroconversion defined as a TPPA titer greater than 1:1280 (Table 3). Consistent with the trends observed in the challenge experiments, transplantation of lesions derived from non‐immunized control rabbits resulted in positive seroconversion (TPPA > 1:40) in recipient rabbits on days 10 and 13 post‐transplantation, respectively. Robust seroconversion was subsequently observed on days 21 and 24, with TPPA titers reaching 1:1280. Following euthanasia, dark‐field microscopic examination of testicular tissues confirmed the presence of actively motile spirochetes, with bacterial burdens of 3.2 × 10^8^ and 9.6 × 10^7^ *T. pallidum*, respectively. Similarly, both recipient rabbits that received lesion transplants from Ad5(wt)‐immunized donors exhibited seroconversion, occurring on days 15 and 19 post‐transplantation, followed by strong seroconversion on days 27 and 32. Dark‐field analysis of testicular tissues revealed *T. pallidum* loads of 1.0 × 10^8^ and 6.4 × 10^7^, respectively, indicating a delayed serological response and reduced bacterial burden compared with the non‐immunized control group. In contrast, among the two recipient rabbits that received lesion transplants from rAd5‐TP0326 (i.n.) immunized donors, one animal (R‐InrAd1) remained negative seroconversion throughout the observation period. The second recipient (R‐InrAd2) developed seroconversion on day 21, with strong seroconversion detected on day 35, and a markedly reduced testicular bacterial burden of 1.6 × 10^7^ *T. pallidum* was recovered. Notably, neither of the two recipient rabbits that received lesion transplants from rAd5‐TP0326 (i.m.) immunized donors (R‐ImrAd1 and R‐ImrAd2) exhibited seroconversion at any time point during the entire monitoring period, demonstrating complete blockade of *T. pallidum* transmission. Overall, the timing of seroconversion and the quantity of *T. pallidum* recovered from the testes in this rabbit transmission model closely mirrored the outcomes observed in the challenge experiments. Groups that exhibited more severe lesion progression, higher local pathogen burdens, and greater histopathological damage in the challenge model consistently showed earlier seroconversion and higher testicular spirochete loads following lesion transplantation (Table 3). These findings demonstrate that intramuscular immunization with rAd5‐TP0326 not only elicits robust protective immunity but also effectively prevents the dissemination and transmission of *T. pallidum* in vaccinated hosts, underscoring its potential significance for public health applications.

**TABLE 3:** Serological Assessment and T. pallidum Harvest in the Rabbit Infectivity Test (RIT) assays.

				Test		
D42 (Chancre)	Donor animal#	DarkField	Recipient animal&	Seroconversion	TPPA (1:1280)	Harvest (Organisms)
Control	Ct1/Ct2	±	R‐Ct1/R‐Ct2	+(Day 10)/+(Day 13)	Day 21/Day 24	3.2×10^8^/9.6×10^7^
rAd5‐Tp0326(i.m)	ImrAd1/ImrAd2	‐/‐	R‐ImrAd1/R‐ImrAd2	‐/‐	‐/‐	‐/‐
rAd5‐Tp0326(i.n)	InrAd1/InrAd2	‐/+	R‐InrAd1/R‐InrAd2	‐/+(Day 21)	‐/Day 35	‐/1.6×10^7^
rAd5‐wt(i.m)	ImAd1/ImAd2	±	R‐ImAd1/R‐ImAd2	+(Day 15)/+(Day 19)	Day 27/Day 32	1.0×10^8^/6.4×10^7^

The symbol ‘ + ’ indicates positive seroconversion (reactive TPPA >1:40). The symbol ‘‐’ indicates negative seroconversion (nonreactive/weakly reactive TPPA (1:40)).

‘#’ Rabbits dermally infected with *T. pallidum*.

‘&’ Rabbits that received testicular injections of the exudate of the ulcer 42 days post‐infection from donor animals.

## 3 Discussion

To advance syphilis and congenital syphilis elimination, a safe and deployable vaccine is urgently needed [31]. While Miller first demonstrated complete protection in rabbits, translational feasibility remains limited [32]. Subsequent *E. coli* derived recombinant antigens (e.g., TprK, Tp0751) lacked confirmed outer membrane protein (OMP) topology and yielded inconsistent protection [33, 34, 35, 36, 37]. Recent structural vaccinology has now identified conserved β‐barrel OMPs with functional extracellular loops (ECLs), such as TP0326 [21]. Although TP0326 shows partial protection, prior subunit and DNA approaches faced methodological constraints [22, 24]. To preserve native conformation, we employed a replication‐defective Ad5 vector delivering full‐length TP0326, circumventing refolding issues of *E. coli* systems and low transfection efficiency of DNA vaccines [38].

In this study, we directly compared an *E. coli* expression platform and a replication‐defective adenoviral vector encoding full‐length TP0326 for their immunogenicity and protective efficacy in murine and rabbit *T. pallidum* challenge models. Within the *E. coli* platform, we evaluated both the full‐length recombinant protein and ECL4 (33 aa, R569‐W601), an extracellular loop implicated in eliciting opsonophagocytic antibodies [39]. All three vaccine regimens induced robust B‐cell responses in both species and showed a Th2‐skewed humoral profile, characterized by high titer, high avidity antibodies and an increased IgG1 proportion. This pattern is consistent with an antibody dominant protective paradigm relevant to preventing mother‐to‐child transmission, supporting adhesion blocking, neutralization, and opsonophagocytosis that may promote sustained spirochete clearance and durable protection in vivo [40, 41, 42, 43].

Compared with controls, antigen specific antibodies elicited by all three regimens bound strongly to full length recombinant TP0326 (rTp0326), yet they differed markedly in reactivity to *T. pallidum* lysates. Antibody titer analysis showed that, in both mice and rabbits, rTP0326 and r*Pf*Trx‐TP0326^ECL4^ induced higher titers than rAd5‐TP0326 (Figures 1b and 2b), whereas rAd5‐TP0326 sera exhibited superior binding to *T. pallidum* lysates (Figures 1d and 2d). These data underscore that syphilis prevention is not simply a matter of generating “more antibodies”, but of inducing high quality functional antibodies that recognize native pathogen conformations particularly surface exposed loops and conformational epitopes. Effective protection likely requires antibodies capable of engaging intact spirochetes in physiologic contexts and triggering effector functions such as opsonophagocytosis, adhesion blockade, and enhanced clearance. Conversely, high titer responses dominated by denatured or linear epitopes may translate poorly in vivo despite robust serological readouts. Therefore, vaccine design should prioritize antigen presentation that preserves natural conformation to reliably elicit functional antibodies capable of recognizing and clearing *T. pallidum*, rather than focusing on antibody magnitude alone [44, 45, 46, 47].

The modest reactivity of rPfTrx‐TP0326^ECL4^ likely reflects ECL4's short length and low native TP0326 abundance. While *E. coli* expressed TP0326 appeared as a discrete ∼94 kDa band (Figure S1c), adenoviral‐mediated expression yielded diffuse bands (Figure S2d), consistent with SEC profiles showing broad 100–440 kDa elution [23]. This, coupled with superior binding of Ad5‐TP0326 sera to native spirochetes, suggests prokaryotic systems may incompletely recapitulate native conformation, potentially due to missing eukaryotic post‐translational modifications like glycosylation [48]. These findings support using eukaryotic platforms to better preserve conformational epitopes critical for functional antibodies [49]. Leveraging the in vitro *T. pallidum* culture system [50, 51, 52], we showed sera from all groups inhibited adhesion, suppressed growth, and enhanced opsonophagocytosis in mice and rabbits [30, 53]. Given TP0326's undefined OMP role, these convergent effects suggest antibodies targeting surface exposed domains disrupt a critical host‐pathogen interface, blocking attachment while promoting immune clearance.

Notably, mouse and rabbit sera exhibited distinct bactericidal profiles: mouse sera were largely bacteriostatic in vitro, whereas rabbit sera mediated robust treponemicidal activity. This divergence likely reflects species specific differences in antibody effector functions and epitope recognition; notably, rabbit antibodies access certain epitopes on human antigens that are less recognizable by murine monoclonals, broadening the effective repertoire [54, 55, 56]. These findings underscore that reliance on a single animal model may inadequately predict functional antibody responses relevant to humans, advocating for integrated models that better approximate human immune effector mechanisms. Comparing opsonophagocytic activity against the homologous Nichols strain versus prevalent SS14‐like isolates revealed a striking pattern. Although the TP0326 immunogen was Nichols‐derived, mouse sera showed comparable activity against both strain types, while rabbit sera exhibited significantly stronger opsonization against SS14‐like isolates (Figures 1i and 2h). Despite TP0326 is high conservation, sequence variation in surface exposed epitopes may introduce “targeting bias” [23]. Crucially, these discordant patterns indicate that sequence conservation alone is insufficient to predict cross strain protection; functional antibody evaluation must incorporate the biological state of test strains (e.g., viability, metabolic activity) to accurately assess efficacy against circulating lineages.

Among animal models, the New Zealand White rabbit remains the gold standard for syphilis research. Decades of foundational work including serial testicular passage, accurate recapitulation of primary and secondary lesions, established reinfection protocols, and characterization of the Jarisch‐Herxheimer reaction have cemented its high pathophysiological relevance and experimental reliability [57, 58, 59, 60]. In this study, we structured efficacy testing into three components: (i) head‐to‐head comparison of vaccine formulations in New Zealand White rabbits; (ii) assessment of protective outcomes following recombinant adenoviral vaccination delivered via different immunization routes; and (iii) subsequent rechallenge to probe resistance to reinfection. Building on our previously established rabbit chancre model and evaluation framework, we used polA quantification in local lesions and serum rapid plasma reagin (RPR) titers to track spirochete clearance kinetics and disease severity, respectively, while the *T. pallidum* particle agglutination (TPPA) test served as a key quality‐control readout confirming successful infection [58, 61].

Following immunization, we challenged rabbits with a dose approximately 100‐fold higher than the estimated clinical infectious dose (ID ≈ 10^2^ CFU).Under ultra‐high challenge, both vaccines induced in vivo opsonophagocytosis (immunofluorescence‐confirmed), mirroring in vitro findings. However, rPfTrx‐TP0326^ECL4^ despite robust in vitro activity failed to attenuate in vivo inflammation (resembling controls), underscoring that in vitro opsonophagocytosis does not predict in vivo efficacy. The rAd5‐TP0326 vaccine consistently outperformed others, yielding no ulceration, minimal lesion diameter, and reduced dissemination. Although sterilizing immunity was unattained, the adenoviral platform markedly constrained early pathogen expansion and accelerated clearance, supporting superior functional potential and motivating evaluation under physiological exposure. Under physiologic dose challenge including an intranasal mucosal immunization arm evaluation of distant organ dissemination was limited by assay sensitivity and the stochastic nature of hematogenous spread. Thus, local assessments at challenge sites served as the primary readout for *T. pallidum* burden (Figures 3c and 5f). We integrated H&E staining, CD68 immunostaining, *T. pallidum* immunofluorescence, and dark‐field microscopy to comprehensively profile local tissue responses.

Notably, one rabbit in the intramuscular rAd5‐TP0326 group achieved complete protection. In the remaining two animals, lesions still developed, but dark‐field microscopy revealed active opsonophagocytosis at the inoculation sites. Together, these findings strongly suggest that antibodies elicited by full length TP0326 can mediate early opsonophagocytic clearance, thereby constraining local spirochete expansion and limiting tissue damage. Consistent with this interpretation, in the gold‐standard Rabbit Infectivity Test (RIT), lesion homogenates from intramuscularly immunized donors failed to establish infection in naïve recipients, supporting complete blockade of transmission and reinfection potential. By comparison, mucosal immunization did not achieve equivalent sterilizing protection under our conditions, but it substantially delayed chancre onset and reduced the likelihood of establishing infection in naïve rabbits, indicating potential value in narrowing the early transmission window, lowering local pathogen burden, and motivating future exploration of genital‐tract mucosal strategies.

Several limitations warrant attention. Our rabbit cellular immunity data were restricted to indirect serum cytokine measurements, lacking antigen‐specific T‐cell profiling. The durability of antibody responses and long‐term protection remains uncharacterized. While prevalent SS14‐like isolates were tested in vitro, cross‐strain protection requires in vivo validation, particularly given the potential for “clonal replacement” under vaccine‐induced selection pressure from polyclonal challenge inocula. Furthermore, intranasal rather than genital mucosal immunization limits inferences regarding transmission blocking efficacy, and consistent sterilizing immunity was not achieved. Finally, the absence of a mother‐to‐child transmission model precludes assessment of vertical transmission risk.

## 4 Conclusion

In this study, we systematically evaluated the protective efficacy of TP0326 across models using multiple expression platforms, immunization routes, and challenge doses. Our data indicate that eukaryotic expression of TP0326 better preserves a native antigen conformation, thereby eliciting higher quality and more functionally active antibody responses and markedly improving local infection control and spirochete clearance. Collectively, these findings support TP0326 as a promising component of an effective syphilis vaccine and provide a clear rationale for future studies addressing durability of immunity, cross‐strain protection, mucosal and mother‐to‐child transmission blocking, and the impact of strain heterogeneity (Figure 8).

**FIGURE 8:**
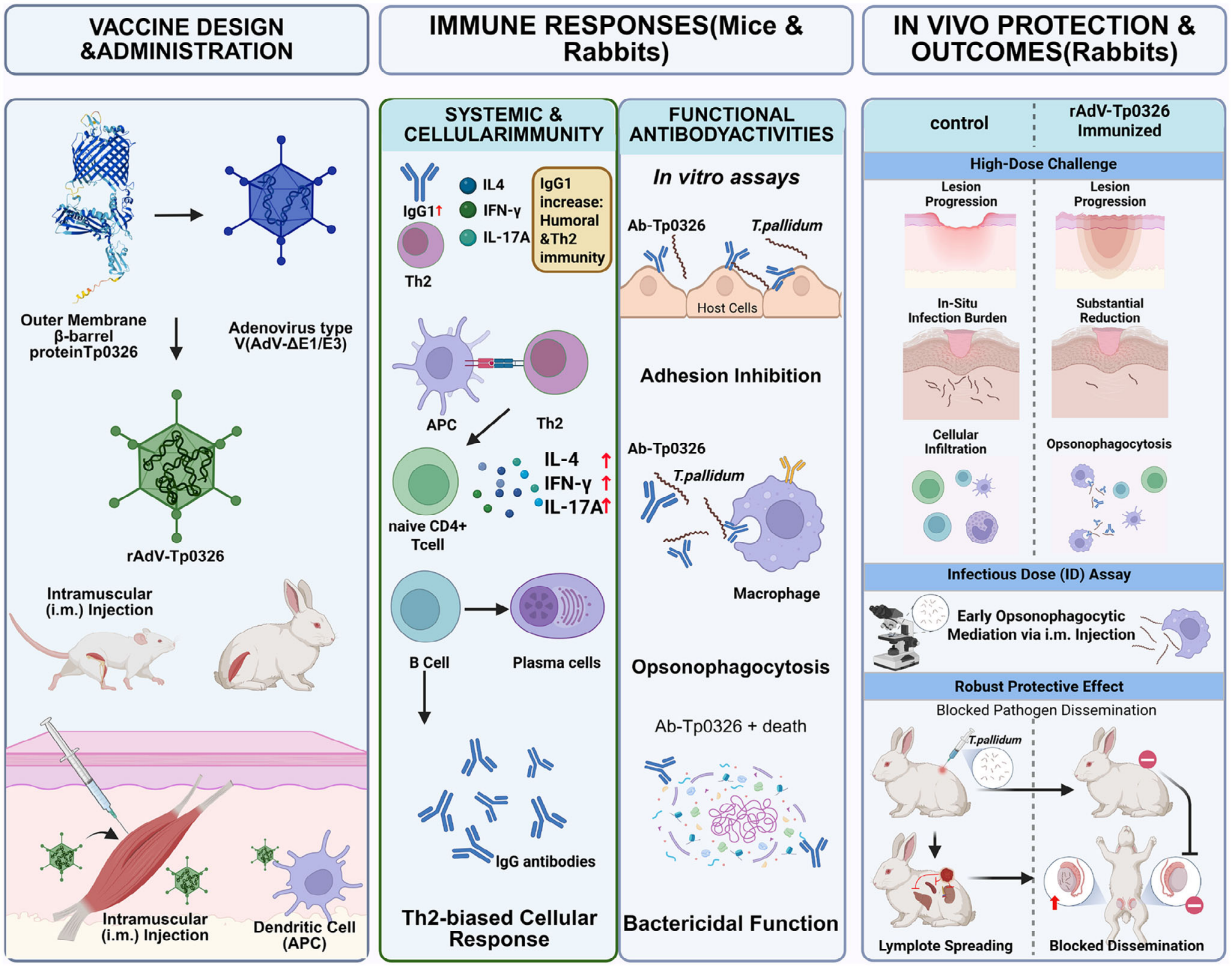
Summary of key findings and proposed mechanisms. Immunization with Tp0326 induced high‐titer functional antibodies in both mice and rabbits. Vaccination promoted cellular infiltration into primary lesions and effectively inhibited the dissemination of *T.pallidum* to distal organs. Notably, transfer of lesion tissue from Tp0326 immunized animals to naïve recipients did not result in infection, confirming the specificity of this protective immunity. We propose that the protective mechanism involves the induction of local antibody production at the primary lesion site, facilitating direct pathogen killing and enhancing spirochete clearance via macrophage phagocytosis. Collectively, these responses prevent the establishment of secondary infections by *T.pallidum*.

## 5 Materials and Methods

### 5.1 Materials

HEK293T cells (ATCC, Cat. No. CRL‐11268) used in the recombinant Ad5‐TP0326 construction assay were cultured in Dulbecco's modified Eagle's medium (DMEM; Gibco) supplemented with 10% fetal bovine serum (FBS) at 37°C in a humidified atmosphere containing 5% CO_2_. For in vitro co‐culture with *T. pallidum*, the rabbit epithelial cell line Sf1Ep was obtained from the American Type Culture Collection (ATCC, Manassas, VA, USA) and maintained in Eagle's minimum essential medium (EMEM; Gibco) supplemented with 10% FBS under the same culture conditions.

Isopropyl‐β‐D‐thiogalactopyranoside (IPTG), His‐tag protein purification kit, and Edu detection kit were purchased from Beyotime Biotechnology (China). Luria–Bertani medium and kanamycin were supplied by Shanghai Sangon Biotechnology Co., Ltd. Freund's adjuvant acquired from Thermo Fisher Scientific (USA). The antibodies used for flow cytometry were listed in Table S1. ELISA kits for Mouse IL‐4, Mouse IL‐17a, Mouse IFN‐γ, Mouse IFN‐γ were procured from RaybioTech Co., Ltd. (China). ELISA kits for Rabbit IL‐4, Rabbit IL‐17a, Rabbit IFN‐γ, were procured from COIBO Co., Ltd. (China). ELISA kits for Mouse IgG1, Mouse IgG2a and Mouse IgG2b were purchased from Elabscience Co., Ltd. (China). HRP—conjugated goat anti‐mouse secondary antibody and HRP‐conjugated goat anti‐rabbit secondary antibody were sourced from Shanghai Sangon Biotechnology Co., Ltd. (China). FITC Anti‐*T. pallidum* antibody was purchased from Abcam (Cambridge, UK; Cat. No. ab20719).

### 5.2 Animals

Female BALB/c mice aged 6–8 weeks and adult male New Zealand White (NZW) rabbits weighing 2.5–3.5 kg were obtained from the Guangdong Medical Laboratory Animal Breeding Center (Guangzhou, China). Mice and Rabbits were maintained under specific pathogen‐free (SPF) conditions at the Animal Experimental Center of South China Agricultural University, with controlled ambient temperature (18°C–20°C) and free access to sterilized drinking water and antibiotic‐free standard chow. Prior to experimental procedures, all animals were confirmed to be seronegative for *T. pallidum* using both treponemal and non‐treponemal serological assays. All animal studies were performed in accordance with institutional animal care and use guidelines and were approved by the Animal Ethics Committee of South China Agricultural University (Approval No. 2021C036).

### 5.3 Protein Expression, Purification, and Storage

Recombinant TP0326 and r*Pf*Trx‐TP0326^ECL4^ proteins were generated as described previously [21, 22], with minor modifications. Briefly, TP0326 containing an N‐terminal 6×His tag was heterologously expressed in Escherichia coli Rosetta (DE3) pLysS competent cells. For construction of the r*Pf*Trx‐TP0326^ECL4^ fusion protein, the r*Pf*Trx‐TP0326^ECL4^ fragment was genetically linked to the C‐terminus of a Pyrococcus furiosus thioredoxin (PfTrx) scaffold and similarly tagged with a 6×His sequence. The fusion construct was expressed in *E. coli* BL21 (DE3) cells cultured at 37°C. Protein expression was induced by the addition of isopropyl β‐D‐1‐thiogalactopyranoside (IPTG) to a final concentration of 0.5 mm when cultures reached an optical density at 600 nm (OD_600_) of approximately 0.4. Recombinant r*Pf*Trx‐TP0326^ECL4^ was purified under native conditions using Ni–NTA affinity chromatography, whereas TP0326 was purified under denaturing conditions. Protein purity exceeded 90%, as assessed by sodium dodecyl sulfate–polyacrylamide gel electrophoresis (SDS–PAGE) and Western blot analysis. Purified proteins were concentrated to 1 mg/mL in phosphate‐buffered saline (PBS) using Amicon centrifugal filter units, rapidly frozen in liquid nitrogen, and stored at −80°C until further use.

### 5.4 Construction of Recombination Adenovirus‐Vector

The regions encoding recombinant proteins were amplified by PCR from *T. pallidum* genomic DNA and cloned into the expression vector pshuttle‐IRES‐rGFP‐1 via restriction sites. The sequences of all constructs were confirmed by DNA sequencing. Correctly sequenced recombinant shuttle plasmid were linearized by the restriction enzyme pmeI and Co‐transformed with the skeleton plasmid pAdeasy‐1 into competent cell E.coli BJ5183. Following 18 to 20 h of incubation the monoclonal cells were extract plasmid and confirmed by Digestion and DNA sequencing. Recombination adenovirus vector were linearized by the restriction enzyme pacI and recovered by agarose gel. Transfections of the linearized plasmid were performed using Lipofectamine LTX(Invitrogen) following 6 to 7 day of incubation and continuously observe the generation of fluorescence and cell pathology. Wild‐type adenovirus type 5 [Ad5 (wt)] was previously prepared and preserved at the Dermatology Hospital of Southern Medical University.

### 5.5 Verification of Viral Protein Expression

HEK293T and Sf1Ep cells were infected with either wild‐type adenovirus (Ad5(wt)) or rAd5‐TP0326 at a multiplicity of infection (MOI) of 10 and incubated for 48 h. GFP fluorescence was monitored using a confocal laser scanning microscope with excitation at 488 nm to evaluate infection efficiency. Following infection, both cells and culture supernatants were harvested by centrifugation. Cell pellets were lysed on ice in RIPA buffer (Beyotime Biotechnology, Shanghai, China), and protein extracts were denatured by heating in 5× SDS sample buffer at 100°C for 10 min. TP0326 expression in rAd5‐TP0326–infected cells was confirmed by Western blot analysis using an anti‐Flag mouse monoclonal antibody (1:5000; Sangon Biotechnology, Shanghai, China) as the primary antibody, followed by incubation with a horseradish peroxidase–conjugated goat anti‐mouse IgG secondary antibody (1:5000; Sangon Biotechnology, Shanghai, China). GAPDH was used as an internal loading control.

### 5.6 Transmission Electron Microscopy

To examine the morphology and structural integrity of Ad5(wt) and rAd5‐TP0326 viral particles, purified virus samples were prepared for transmission electron microscopy (TEM) analysis. Aliquots of CsCl‐purified viral suspensions were applied onto carbon‐coated copper grids and allowed to adsorb for 5–10 min at room temperature. Excess liquid was gently removed using filter paper, and the grids were negatively stained with 2% (w/v) phosphotungstic acid (pH 7.0) for 30–60 s. After air‐drying, the grids were examined using a transmission electron microscope operated at an appropriate accelerating voltage. Representative images were acquired to assess viral particle size, morphology, and capsid integrity.

### 5.7 TCID50 Assay

The tissue culture infectious dose 50% (TCID_50_) of the virus was determined using an endpoint dilution assay on HEK293T cells. Briefly, HEK293T cells were seeded into 96‐well plates and cultured overnight to reach approximately 80–90% confluence. Viral samples were serially diluted 10‐fold in serum‐free Dulbecco's modified Eagle medium (DMEM). Each dilution was added to the cells in multiple replicates (8 wells per dilution), while control wells received medium only. After adsorption at 37°C for 2 h, the inoculum was removed and replaced with fresh DMEM supplemented with 5% fetal bovine serum. Cells were then incubated at 37°C in a humidified atmosphere with 5% CO2 and observed daily for virus‐induced cytopathic effects (CPE) for up to 5 days. Wells were scored as positive or negative based on the presence of CPE, and the TCID_50_ was calculated using the Reed–Muench method. Viral titers were expressed as TCID_50_ per milliliter (TCID_50_/mL).

### 5.8 Mouse Vaccination Procedure

Five female BALB/c mice were randomly assigned to each experimental group. Mice in the r*Pf*Trx‐TP0326^ECL4^ protein group and the rTP0326 protein group were immunized intraperitoneally with 200 µL of complete Freund's adjuvant (CFA) emulsified with 50 µg of antigen on Day 0, followed by a booster immunization on Day 21 and Day 42 using incomplete Freund's adjuvant (IFA) containing 25 µg of antigen. Animals in the rAd5‐TP0326 and Ad5(wt) groups received an intramuscular injection of 1×10^7^ plaque‐forming units (PFU) of rAd5‐TP0326 or Ad5(wt), respectively, in a total volume of 50 µL on Day 0, with a second identical viral immunization administered on Day 21. Mice in the control group were injected intramuscularly with 50 µL of sterile saline. Peripheral blood samples were collected on Days 21 and 42 and 56(recombinant protein group) for serological detection of anti‐rTP0326 antibodies. On day 42 and day 63, corresponding to three weeks after the final immunization, mice were euthanized, and spleens were harvested for subsequent analysis of T‐cell responses.

### 5.9 Rabbit Vaccination Procedure

Three adult male New Zealand White (NZW) rabbits were included in each experimental group and immunized on Days 0 and 21. Rabbits assigned to the r*Pf*Trx‐TP0326^ECL4^ and rTP0326 protein groups received a subcutaneous injection at the nape of the neck of 800 µL of complete Freund's adjuvant (CFA) containing 400 µg of antigen for primary immunization, followed by a booster dose of 800 µL of incomplete Freund's adjuvant (IFA) containing 200 µg of antigen administered at the same site. Animals in the rAd5‐TP0326 and Ad5(wt) groups were immunized intramuscularly with 1 × 10^8^ PFU of rAd5‐TP0326 or Ad5(wt), respectively, in a total volume of 1 mL, with the viral immunization repeated 21 days later. Blood samples were collected on Days 21 and 42 for serological evaluation of anti‐rTP0326 antibody responses.

### 5.10 Animal Blood Collection and Splenocyte Isolation

In mice, interim blood samples were obtained by tail vein bleeding, while terminal blood collection was performed by cardiac puncture under anesthesia. For rabbits, blood samples were collected via the marginal ear vein. Collected blood was allowed to clot at room temperature for 30 min and subsequently centrifuged at 3,000 rpm for 10 min to separate serum, which was stored at −80°C until further analysis.

Spleens were collected from five mice per group on Day 42 and processed individually. Each spleen was mechanically dissociated through a 70‐µm cell strainer (Fisher Scientific) into 5 mL of complete RPMI medium (RPMI 1640 supplemented with 10% fetal bovine serum and 1% penicillin–streptomycin). Red blood cells were removed using ACK lysis buffer, after which cell suspensions were filtered and washed. Cell viability and total cell counts were determined by trypan blue exclusion using a hemocytometer.

### 5.11 Serum Antibody Responses to Vaccination

Antibody responses against rTP0326 were evaluated by enzyme‐linked immunosorbent assay (ELISA). Briefly, 96‐well plates were coated overnight at 4°C with rTP0326 protein at a concentration of 1 µg/mL diluted in carbonate–bicarbonate buffer (pH 9.6). Plates were washed twice with TBST (TBS containing 0.05% Tween‐20) and subsequently blocked with 5% skim milk prepared in TBST for 90 min at 37°C. Serial twofold dilutions of mouse or rabbit sera (ranging from 1:2,000 to 1:1,638,400) were added to the plates and incubated for 1 h at 37°C. After washing twice with TBST, bound antibodies were detected using horseradish peroxidase (HRP)‐conjugated goat anti‐mouse IgG or HRP‐conjugated goat anti‐rabbit IgG secondary antibodies (both diluted 1:8,000; Sangon Biotech, Shanghai, China) for 45 min at 37°C. Signal development was achieved using tetramethylbenzidine (TMB) substrate solution (Bioss, Cat. No. C04‐03002) and terminated with stop solution. Absorbance was recorded at 450 nm within 20 min using a Varioskan LUX multimode microplate reader (Thermo Fisher Scientific).

### 5.12 Cellular Proliferation Assay

Spleens from BALB/c mice were isolated and processed into single‐cell suspensions, which were seeded into 24‐well plates at a density of 1 × 10^6^ cells per well. Cells were stimulated with 10 µg/mL rTP0326 and incubated at 37°C for 24 h. Subsequently, 300 µL of EdU‐containing medium was added to each well, followed by an additional 2 h incubation. Cells were then fixed with 4% paraformaldehyde for 30 min, treated with 2 mg/mL glycine for 5 min, and permeabilized using 0.5% Triton X‐100 for 10 min.

Incorporation of EdU was detected using a commercial EdU assay kit (Sangon Biotech, Shanghai, China) according to the supplied protocol. Cell nuclei were counterstained with Hoechst 33342 for 20–30 min. EdU‐positive cells were visualized and captured using fluorescence microscopy.

### 5.13 Cytokine Secretion Assays

To assess antigen‐induced cytokine production, splenocytes from BALB/c mice were plated at 1 × 10^6^ cells per well in 24‐well plates and stimulated with rTP0326 at a final concentration of 10 µg/mL for 48 h. Following incubation, culture supernatants were harvested and analyzed for IL‐2, IL‐17A, TNF‐α, and IFN‐γ levels using commercially available ELISA kits (Absin, Shanghai, China), following the manufacturers’ instructions. Cytokine concentrations were determined based on standard curves and are presented as pg/mL.

### 5.14 Flow Cytometry

Intracellular cytokine staining (ICS) was conducted to characterize vaccine‐elicited T‐cell phenotypes. Freshly prepared mouse splenocytes were seeded at a density of 5 × 10^6^ cells per well and incubated in complete RPMI 1640 medium supplemented with either rTP0326 (stimulated condition) for 4 h at 37°C in the presence of an Fc receptor–blocking reagent. After stimulation, cells were labeled with antibodies against surface markers, including CD3, CD4, CD8, CD25, CD62L, and CD69. Cells were subsequently fixed and permeabilized prior to intracellular staining for cytokines and cytotoxic molecules, including IL‐4, IFN‐γ, IL‐17, perforin, and granzyme B, using fluorochrome‐conjugated antibodies. Nonviable cells were excluded using the fixable viability dye eFluor 506 (Thermo Fisher Scientific), and nuclear staining was performed with DAPI. Flow cytometric data were collected using a BD FACSCelesta instrument and analyzed with FlowJo software (BD Biosciences).

### 5.15 Determination of IgG Subclass Responses by ELISA

The IgG subclass distribution (IgG1, IgG2a, and IgG2b) in mouse immune sera was determined using enzyme‐linked immunosorbent assay (ELISA). All IgG subclass–specific ELISA kits (Elabscience, Wuhan, China) were used according to the manufacturer's protocols. ELISA plates were coated overnight at 4°C with the indicated antigen at an appropriate concentration diluted in coating buffer. Plates were then blocked with blocking buffer provided by the kit to prevent nonspecific binding. Mouse serum samples were diluted according to the manufacturer's instructions and added to the wells, followed by incubation at 37°C for the recommended duration. After washing to remove unbound antibodies, horseradish peroxidase HRP‐conjugated detection antibodies specific for mouse IgG1, IgG2a, or IgG2b were applied. Color development was achieved using tetramethylbenzidine (TMB) substrate and stopped with stop solution. The absorbance was measured at 450 nm using a microplate reader. IgG subclass levels were quantified based on optical density values, and the IgG1/IgG2a ratio was calculated to evaluate Th1‐ or Th2‐biased immune responses elicited by different immunization strategies.

### 5.16 IgG Purification

Total IgG was purified from rabbit serum samples using a Protein A Sepharose 4FF affinity chromatography kit (Sangon Biotech, Shanghai, China), according to the manufacturer's instructions. Samples were equilibrated to binding conditions (0.15 m NaCl, 20 mM Na_2_HPO_4_, pH 7.0–7.5) by dilution or buffer exchange and clarified by centrifugation or filtration through a 0.22 µm membrane prior to loading. Protein A Sepharose 4FF resin was equilibrated with five column volumes of binding/wash buffer and incubated with the prepared samples to allow IgG binding. Unbound proteins were removed by extensive washing with binding/wash buffer until a stable baseline was achieved. Bound IgG was subsequently eluted using 0.1 m glycine or citric acid buffer (pH 3.0). Eluted fractions were immediately neutralized with 1 m Tris‐HCl (pH 8.5) at a ratio of 1:10 (v/v) to preserve antibody integrity. Purified IgG fractions were pooled and, when necessary, buffer‐exchanged into phosphate‐buffered saline (PBS) for downstream applications. The concentration and purity of IgG were assessed by standard protein quantification methods and SDS–PAGE analysis. The Protein A resin was regenerated and stored in PBS containing 20% ethanol at 2°C–8°C for subsequent reuse.

### 5.17 Serum Reactivity Assay

Western blotting was performed to evaluate the reactivity of post‐immunization mouse sera against recombinant TP0326 (rTP0326) protein and *T. pallidum* lysates. Fresh, viable *T. pallidum* organisms were obtained from rabbit passage experiments. A total of 1 × 10^7^ treponemes were harvested and lysed to prepare whole‐cell lysates. *T. pallidum* lysates and rTp0326 protein at varying concentrations were resolved by SDS–PAGE and transferred onto PVDF membranes. Membranes were blocked and incubated with mouse or rabbit sera collected after immunization with rAd5‐TP0326, rTP0326 protein, rPfTrx‐TP0326^ECL4^, or Ad5(wt), which were used as primary antibodies. After washing, horseradish peroxidase (HRP)‐conjugated goat anti‐mouse or anti‐rabbit IgG secondary antibodies were used at a dilution of 1:5000 (Shanghai Sangon Biotechnology Co., Ltd., China). Immunoreactive bands were visualized using an enhanced chemiluminescence (ECL) detection system.

### 5.18 Assessment of Functional Activity Using In Vitro Cultivated *T. pallidum*

Cottontail rabbit epithelial (Sf1Ep) cells were seeded into 48‐well plates at a density of 1 × 10^3^ cells per well and incubated at 37°C. On the following day, culture wells were rinsed once with *T. pallidum* culture medium 2 (TpCM‐2) that had been pre‐equilibrated under microaerobic conditions (1.5% O2, 3.5% CO2, and 95% N2). Subsequently, 1 mL of fresh TpCM‐2 was added to each well, and plates were maintained under microaerobic conditions for at least 3 h. Freshly harvested *T. pallidum* organisms (1× 10^5^ per well) were then introduced into each well together with heat‐inactivated normal mouse or rabbit serum, infected‐rabbit serum, or sera collected from mice and rabbits immunized with r*Pf*Trx‐TP0326^ECL4^, rTP0326, Ad5 (wt), or rAd5‐TP0326. Co‐cultures were maintained for 7 days under microaerobic conditions. Following incubation, culture supernatants were collected and reserved for subsequent dark‐field microscopy (DFM) enumeration. To recover cell‐associated spirochetes, wells were washed once with 200 µL of trypsin‐EDTA to remove residual TpCM‐2 medium. An additional 100 µL of trypsin‐EDTA was added to each well, followed by incubation at 37°C for 5 min. *T. pallidum* organisms released from the epithelial cells were then collected into separate 2‐mL conical tubes. Both the supernatant and the trypsinized (cell‐associated) fractions were centrifuged at 1,000 rpm for 5 min prior to DFM enumeration.

### 5.19 Opsonophagocytosis Assays

Opsonophagocytosis assays were performed as previously described in a published study [29] to evaluate the Fc receptor–dependent opsonophagocytic activity of vaccine‐induced antibodies. Rabbit peritoneal macrophages were elicited by intraperitoneal injection of 10% proteose peptone, harvested in ice‐cold PBS containing EDTA, and plated onto poly‐D‐lysine–coated glass chamber slides at a density of 1 × 10^5^ cells per well. After incubation at 37°C for 2 h to allow adherence, non‐adherent cells were removed by washing with DMEM. Murine bone marrow–derived macrophages (BMDMs) were generated from femurs and tibias of Balb/c mice, differentiated in DMEM supplemented with macrophage colony‐stimulating factor (M‐CSF) for 7 days, and seeded onto 8‐well chamber slides at the same density overnight in a humidified 37°C incubator with 5% CO_2_. For opsonophagocytosis, freshly harvested *T. pallidum* Nichols strain and two SS14 strains were diluted in DMEM and incubated with heat‐inactivated mouse or rabbit sera (56°C for 30 min) from control and immunized groups at a final concentration of 5% (v/v) for 2 h at room temperature to allow antibody‐mediated opsonization. The opsonized spirochetes were then added to rabbit macrophages or murine BMDMs at an MOI of 10:1 and incubated for 4 h at 37°C in triplicate. After incubation, supernatants were removed, and cells were washed three times with PBS to eliminate extracellular spirochetes.

### 5.20 Immunofluorescence Staining and Quantification

Cells were fixed with 2% paraformaldehyde for 10 min at room temperature, permeabilized with 0.01% Triton X‐100, and blocked with 5% bovine serum albumin (BSA) in PBS for 1 h. Internalized *T. pallidum* was detected by incubation with a FITC Anti‐*T. pallidum* antibody (Abcam, Cambridge, UK; Cat. No. ab20719) at a 1:200 dilution. Then, actin cytoskeletons were stained with Phalloidin‐TRITC 555 for 20 min, and nuclei were stained with DAPI for 10 min; subsequently, imaging was performed via laser confocal microscopy (Nikon, Japan, A1+).

Phagocytosis was assessed in a blinded manner by fluorescence microscopy. Images of at least 100 macrophages per well were acquired and analyzed. The phagocytic rate was calculated as the number of internalized spirochetes per 100 observed spirochetes. Data were compared among different serum treatment groups to evaluate antibody‐mediated opsonophagocytic activity against both Nichols and SS14 strains.

### 5.21 Challenge Experiments

The *T. pallidum* Nichols strain was maintained at the Dermatology Hospital of Southern Medical University and routinely propagated by intratesticular passage in New Zealand White (NZW) rabbits as described previously [58]. For the high‐dose challenge study, freshly harvested *T. pallidum* organisms were inoculated intradermally into the dorsal skin of rabbits at eight distinct sites, with a dose of 1 × 10^5^ organisms per site. Animals were examined daily for the appearance and progression of skin lesions. Peripheral blood samples were obtained from each rabbit at 7 day intervals, and serum was analyzed for *T. pallidum* particle agglutination (TPPA) and rapid plasma reagin (RPR) titers. At 21 days post‐infection, rabbits were euthanized, and lesion tissues were collected for DNA extraction and determination of *T. pallidum* burden. For histological evaluation, excised lesion samples were fixed in 4% paraformaldehyde at room temperature.

To assess the presence of viable spirochetes, lesion exudates were obtained by gently swabbing the lesion surface with saline‐soaked cotton swabs. *T. pallidum* recovered from each inoculation site was examined by dark‐field microscopy, and live organisms were counted across five microscopic fields per sample.

In the second immunization–challenge experiment, rabbits were immunized with PBS, rAd5‐TP0326 via intranasal (i.n.) or intramuscular (i.m.) routes, or Ad5(wt), using the same immunization protocol as in the initial experiment. Three weeks after the final immunization, blood samples were collected to measure rTP0326‐specific antibody titers. Rabbits were subsequently challenged intradermally at eight dorsal sites with 10^2^ *T. pallidum* organisms per site. Lesion formation was monitored daily, and blood samples were collected weekly for TPPA and RPR testing. At 21 days post‐challenge, two lesions per rabbit were harvested for DNA extraction and quantitative PCR (qPCR) analysis of *T. pallidum* load, while an additional lesion from each animal was collected for histopathological examination and fixed in 4% paraformaldehyde at room temperature.

### 5.22 DNA Extraction and Purification

Genomic DNA was isolated from tissues collected from *T. pallidum*‐challenged rabbits. Following euthanasia and tissue collection, samples from chancres, heart, liver, spleen, lung, and kidney were digested with proteinase K. DNA extraction was performed immediately using the DNeasy Blood & Tissue Kit (QIAGEN, Hilden, Germany; 69506) in accordance with the manufacturer's instructions.

### 5.23 Real‐Time Quantitative Polymerase Chain Reaction (RT‐qPCR)

Quantification of *T. pallidum* DNA was carried out using a TaqMan probe‐based quantitative PCR assay targeting the polA gene as described previously [58]. Each 25 µL reaction consisted of 12.5 µL of 2 × TaqMan Gene Expression Master Mix (Thermo Fisher Scientific), 2.5 µL of a 2.5 µm forward and reverse primer mixture, 2.5 µL of a 2 µm FAM‐labeled probe, 1 µL of 50 mm MgCl_2_, 4 µL of nuclease‐free water, and 2.5 µL of template DNA. Amplification was performed on a CFX96 Real‐Time PCR System (Bio‐Rad) with an initial denaturation step at 95°C for 10 min, followed by 40 cycles of 95°C for 15 s and 60°C for 1 min. Absolute copy numbers were calculated using a standard curve generated from plasmids containing the polA gene sequence.

### 5.24 Histology

At 21 days post‐challenge, biopsy punch specimens (2 mm in diameter) were obtained from a single lesion of each rabbit. Tissue samples were fixed in formalin, embedded in paraffin, bisected, and sectioned vertically at a thickness of 4 µm. Sections were stained with hematoxylin and eosin (H&E) and examined under light microscopy to evaluate immune cell infiltration, including lymphocytes, neutrophils, dendritic cells, and differentiated B cells. Quantitative assessment was conducted in a blinded manner at 400× magnification by counting cells in five randomly selected fields from two independent sections per sample. In addition, immunohistochemical staining for CD68 was performed to assess macrophage infiltration. Immunofluorescence staining was also used to visualize the morphology and spatial distribution of *T. pallidum* within lesion tissues by confocal laser scanning microscopy.

### 5.25 Rabbit infectivity test (RIT)

To further evaluate whether rAd5‐TP0326 immunization limited the dissemination of *T. pallidum*, a rabbit infectivity test (RIT) was conducted. At 42 days following challenge with 1 × 10^2^ *T. pallidum* organisms, skin lesions were collected from two NZW rabbits per group, including the control group, rAd5‐TP0326 (intranasal, i.n.) group, Ad5(wt) group, and rAd5‐TP0326 (intramuscular, i.m.) group. Lesion tissues were aseptically excised, homogenized in sterile saline, and prepared as inocula. Tissue homogenates were subsequently inoculated intratesticularly into recipient rabbits according to established RIT protocols [62]. Recipient animals were monitored longitudinally and subjected to serological testing. Seroconversion was defined as a TPPA titer exceeding 1:40. Rabbits were humanely euthanized when TPPA titers reached 1:1280, and testes were collected for further analysis. Testicular extracts were examined by dark‐field microscopy to detect and quantify viable *T. pallidum* organisms.

### 5.26 Statistical Analysis

Statistical analysis was performed using SPSS version 25.0 for Windows (IBMCorp., Armonk, N.Y., USA), GraphPad Prism version 8.3.0 for Windows (GraphPad Software, Inc., San Diego, California USA), and R version 4.2.3 for Windows (R Foundation for Statistical Computing, Vienna, Austria). Data are presented as mean ± standard deviation (SD). For comparisons among three or more groups, the one‐way analysis of variance (ANOVA) was applied, and Tukey's multiple comparison test was used for pairwise comparisons. For comparisons of the proportion of ulcers at inoculation sites between groups, the Chi‐square test was used with the Bonferroni method for pairwise comparison. A p‐value < 0.05 was considered statistically significant.

## Author Contributions

Y.J. conceived and designed the study, selected experimental methods, provided key materials, animal models, and technical platforms, and drafted the initial manuscript. Y.J and X.L. performed experiments and collected data. L.H. and X.Z. contributed to experimental work, figure generation, and data visualization. X.L. generated figures and contributed to manuscript revision and proofreading. W.K. provided scientific supervision and assisted in manuscript revision and editing. W.K. oversaw project management and scientific direction and contributed to manuscript revision, editing, and final approval. All authors reviewed and approved the final manuscript.

## Ethics Statement

All animal experiments were approved by the Institutional Animal Care and Use Committee of South China Agricultural University (Approval No. 2021c036).

## Conflicts of Interest

The authors declare no conflicts of interest.

## Supporting information




**Supporting file 1**: advs78078‐sup‐0001‐SuppMat.docx.

**Supporting file 2**: advs78078‐sup‐0002‐VideoS1.mp4.

**Supporting file 3**: advs78078‐sup‐0003‐VideoS2.mp4.

## Data Availability

Data sharing not applicable to this article as no datasets were generated or analysed during the current study.
