# Supplementary material for: An Adenoviral‐Vectored Tp0326 Vaccine Elicits Robust Functional Antibodies to Prevent Treponema pallidum Dissemination in a Rabbit Model
Source: Adv Sci (Weinh). 2026 Sep 28:e78078. Online ahead of print. doi: 10.1002/advs.78078 (PMC13620261; doi:10.1002/advs.78078)
Supplement: Supplementary file 1 [file ADVS-9999-e78078-s003.docx]

**Supplementary Materials**

**Supplementary Figure 1. The full-length recombinant TP0326 protein and the r*Pf*Trx-TP0326^ECL4^ fusion protein were expressed separately using the Escherichia coli expression system.**

**(a)** Identification of the rpfTrx-TP0326^ECL4^ fusion protein purified by the Ni-NAT resin column using Coomassie Brilliant Blue staining. **(b)** Identification of the rpfTrx-TP0326^ECL4^ fusion protein purified by the Ni-NAT resin column via Western blot analysis. **(c)** Identification of the rTP0326 recombinant protein purified by the Ni-NAT resin column using Coomassie Brilliant Blue staining. **(d)** Identification of the rTP0326 recombinant protein purified by the Ni-NAT resin column via Western blot analysis.

**Supplementary Figure 2. Preparation and characterization of recombinant defective adenovirus of TP0326.**

**(a)** Restriction enzyme digestion analysis for the identification of pAdeasy-IRES-TP0326-GFP Vector. **(b)** Infection of HEK293T cells with recombinant adenovirus. **(c)** Infection of Sf1ep cells with recombinant adenovirus. **(d)** Blots were probed with anti-Flag and Goat-anti-mouse-HRP antibody to confirm the expression. **(e)** Characterization of wild-type and recombinant adenoviruses by transmission electron microscopy.

**Supplementary Figure 3. The *in vitro* opsonophagocytic activity of serum antibodies from vaccine-immunized mice against the Nichols reference strain and the clinical SS14L strain.**

(a) The opsonophagocytic function of immune sera antibodies from different vaccination regimens against the Nichols reference strain was detected by immunofluorescence. **(b)** The opsonophagocytic function of immune sera antibodies from different vaccination regimens against the clinical SS14L strain (smudh02_SS14) was detected by immunofluorescence. **(c)** The opsonophagocytic function of immune sera antibodies from different vaccination regimens against the clinical SS14L strain (smudh04_SS14) was detected by immunofluorescence.

**Supplementary Figure 4. Differential activation and functional polarization of T cell subsets following TP0326 vaccination in mice.**

**(a)** Representative flow cytometry gating strategy for surface activation markers on CD4^+^T cells. **(b)**Gating strategy for intracellular cytokine staining to assess CD4^+^T helper cell polarization. **(c)** Gating strategy for intracellular staining of CD8^+^T cells expressing granzyme B and perforin. **(d)** Quantification of CD25^+^, CD62L^+^, and CD69^+^CD4^+^T cells, intracellular staining CD4^+^T cells expressing IL-4^+^, IFN-γ^+^, L-17A^+^ and CD8^+^T cells expressing granzyme B and perforin following immunization. **(e)** T cell proliferation assessed via EdU incorporation assay following in vitro antigen stimulation. Both vaccine groups showed enhanced T cell proliferation compared to controls, indicating effective T cell priming. **(f)** The concentrations of interleukin-2 (IL-2), interleukin-17A (IL-17A), Tumor necrosis factor(TNF-α) and interferon-gamma (IFN-γ) in the sera of each vaccine immunization group. **(g)** The concentrations of IgG2b in the sera of mice in each vaccine group at the immunological endpoint. n.s.: no significance.

**Supplementary Figure 5. The *in vitro* opsonophagocytic activity of serum antibodies from vaccine-immunized rabbits against the Nichols reference strain and the clinical SS14L strain.**

**(a)** The opsonophagocytic function of immune sera antibodies from different vaccination regimens against the Nichols reference strain was detected by immunofluorescence. **(b)** The opsonophagocytic function of immune sera antibodies from different vaccination regimens against the clinical SS14L strain (smudh02_SS14) was detected by immunofluorescence. **(c)** The opsonophagocytic function of immune sera antibodies from different vaccination regimens against the clinical SS14L strain (smudh04_SS14) was detected by immunofluorescence.

**Supplementary Figure 6. Characterization of vaccine-induced humoral and cellular immune responses prior to pathogen challenge.**

**(a)** Purify the IgG in rabbit sera from different vaccineimmunization groups using Protein A resin. **(b)** Serum cytokine levels measured by ELISA prior to *T. pallidum* challenge at doses of 1 × 10^5^ organisms.

**Supplementary Figure 7. Pathological phenotypic alterations in the intradermal regions of rabbit backs following vaccination and subsequent high-dose *T. pallidum* challenge (1 × 10^5^ organisms per site).**

**(a)** Chancres on the dorsal skin of rabbits in different immunization groups and the result of dark-field examination.

**Supplementary Figure 8. Serum cytokine levels measured by ELISA prior to *T. pallidum* challenge at doses of 1 × 10^2^ organisms.**

**Supplementary Figure 9. Pathological phenotypic alterations in the intradermal regions of rabbit backs following vaccination and subsequent low-dose *T. pallidum* challenge (1 × 10^2^ organisms per site).**

**(a)** Chancres on the dorsal skin of rabbits in different immunization groups and the result of dark-field examination.

**Supplementary Table 1. Antibodies used for flow cytometry**

| **Antibody** | **Clone** |
| --- | --- |
| Alexa Fluor® 488 anti-mouse CD45 | 30-F11 |
| PerCP/Cyanine5.5 anti-mouse CD3ε | 145-2C11 |
| Brilliant Violet 510™ anti-mouse CD4 | RM4-5 |
| Alexa Fluor® 647 anti-mouse CD8a | 53-6.7 |
| APC/Cyanine7 anti-mouse CD25 | 3C7 |
| PE anti-mouse CD69 | H1.2F3 |
| Alexa Fluor® 700 anti-mouse CD62L | MEL-14 |
| PE anti-mouse IL-4 | 11B11 |
| Alexa Fluor® 647 anti-mouse IL-17A | TC11-18H10.1 |
| Alexa Fluor® 700 anti-mouse IFN-γ | XMG1.2 |
| PE anti-mouse Perforin | S16009A |
| Brilliant Violet 421™ anti-human/mouse Granzyme B Recombinant | QA18A28 |

All antibodies were purchased from BioLegend, San Diego, CA, USA.

**Supplementary Table 2. Lesion Progression in Different TP0326 Vaccine Groups at Days 7, 14, and 21 Following Challenge with a 1 × 10^5^ Infectious Dose**

**Supplementary Table 3. Longitudinal Monitoring of Lesion Progression Following Challenge with a 1 × 10^2^ Infectious Dose in Rabbits Immunized via Different Routes with Recombinant Adenoviral Vaccines**
